# Hypoxia‐inducible factor‐2α directly promotes *BCRP* expression and mediates the resistance of ovarian cancer stem cells to adriamycin

**DOI:** 10.1002/1878-0261.12419

**Published:** 2019-01-14

**Authors:** Miao He, Huizhe Wu, Qian Jiang, Yinuo Liu, Li Han, Yuanyuan Yan, Binbin Wei, Fangxiao Liu, Xiaolan Deng, Huiying Chen, Lin Zhao, Min Wang, Xin Wu, Weifan Yao, Haishan Zhao, Jianjun Chen, Minjie Wei

**Affiliations:** ^1^ Department of Pharmacology School of Pharmacy China Medical University Shenyang China; ^2^ Liaoning Key Laboratory of molecular targeted anti‐tumor drug development and evaluation Shenyang China; ^3^ Department of gynaecology Shengjing Hospital of China Medical University Shenyang China; ^4^ Department of gynaecology The First Affiliated Hospital of China Medical University Shenyang China; ^5^ Department of Cancer Biology University of Cincinnati Cincinnati OH USA

**Keywords:** adriamycin, drug resistance, hypoxia, hypoxia‐inducible factor‐2α, ovarian cancer stem cells

## Abstract

Ovarian cancer stem cells (OCSCs) are sources of tumor chemoresistance and recurrence. A hypoxic microenvironment contributes to the chemoresistance of cancer stem cells (CSCs), but the underlying mechanism is not fully understood yet. Here, we show that increased HIF‐2α expression is associated with enhanced stemness of OCSCs and poor outcomes in ovarian cancer patients. OVCAR‐3 and CAOV‐3 sphere‐forming (OVCAR‐3 S and CAOV‐3 S) cells with OCSC‐like properties showed strong resistance to adriamycin (ADR). Hypoxia (1% O_2_) induced high expression of both HIF‐1α and especially HIF‐2α, and increased the resistance of OVCAR‐3 S and CAOV‐3 S cells to ADR. Notably, treatment with ADR further increased the expression of HIF‐2α, but not that of HIF‐1α. Knockdown of *HIF‐2*α expression substantially attenuated the resistance of OVCAR‐3 S and CAOV‐3 S cells to ADR, and the *HIF‐2*α overexpression had the opposite effect. Furthermore, in mouse models xenografted with OCSCs, *HIF‐2*α depletion significantly inhibited tumor growth and sensitized OCSCs to ADR *in vivo*. Mechanistically, HIF‐2α directly promotes transcription/expression of *BCRP*, a gene encoding a transporter protein responsible for pumping drugs (e.g., ADR) out of cells, which in turn increases drug resistance due to increased drug transportation. Collectively, our studies reveal a novel drug‐resistant mechanism in ovarian cancer by which hypoxia (and ADR treatment)‐induced HIF‐2α overexpression endows OCSCs with resistance to ADR by promoting *BCRP* expression and ADR transportation. Therefore, targeting the HIF‐2α/BCRP axis holds therapeutic potential for treating drug‐resistant ovarian cancer.

**Abbreviations**
ABCG2: ATP‐binding cassette subfamily G member 2
ADR: adriamycin
ALDH1A1: aldehyde dehydrogenase isoform 1A1
BCRP: breast cancer resistance protein
CSCs: cancer stem cells
DDP: cisplatin
HIF‐2α: hypoxia‐inducible factor‐2α
HREs: hypoxia‐response elements
MDR: multidrug resistance
MFI: mean fluorescence intensity
MX: mitoxantrone
OCSCs: ovarian cancer stem cells
PTX: paclitaxel
shRNA: short hairpin RNA
TCGA: the Cancer Genome Atlas
VP‐16: etoposide

## 1 Introduction

Ovarian cancer is the most lethal gynecological malignancy and one of the leading causes of cancer‐related mortality in women in the USA, resulting in an estimated 22 440 new cases and 14 080 deaths in 2017 according to the American Cancer Society (Siegel *et al*., 2017). It was estimated that about 52 100 new cases of ovarian cancer were diagnosed and 22 500 women died of ovarian cancer in China in 2015 (Chen *et al*., 2016). Although most ovarian tumors initially respond to chemotherapy, refractory tumors often emerge as a result of expansion of clones with either innate or acquired resistance, which then ultimately develop into recurrent tumors (Ottevanger, 2017). It is increasingly evident that heterogeneous ovarian cancers contain a subpopulation of cancer stem cells (CSCs) with enhanced self‐renewal, differentiation, and chemoresistance capabilities (Jung *et al*., 2016). These ovarian CSCs (OCSCs), with their increased chemoresistance, are believed to contribute to tumor maintenance and recurrence; therefore, targeting OCSCs may provide a more effective novel therapeutic strategy for ovarian cancer treatment.

Hypoxia within tumors is emerging as a critical niche for CSCs. The microenvironment associated with hypoxia contributes to chemoresistance of the tumor through the generation of CSCs (Baran and Konopleva, 2017; Maugeri‐Sacca *et al*., 2011). Hypoxia‐inducible factor‐2α (HIF‐2α), a new recently identified member of the HIF family, complexes with HIF‐1β to form a heterodimer that binds to the conserved hypoxia‐response elements (HREs) in the promoters of targeted genes as a transcription factor under hypoxic conditions, similar to HIF‐1α; HIF‐2α is degraded in an oxygen‐dependent manner via the von Hippel‐Lindau protein pathway (Koh and Powis, 2012). Previous studies have shown that HIF‐1 is associated with the chemotherapy failure in cancer cells including ovarian cancer (Ai *et al*., 2016; Heddleston *et al*., 2010). Accumulating evidence in recent years suggests that HIF‐2 plays important roles in the chemoresistance of tumor cells, especially in promoting a more stem‐like phenotype in CSCs (Pietras *et al*., 2009; Zhao *et al*., 2014). It was reported that silencing HIF‐2α expression reduced *in vitro* self‐renewal ability and *in vivo* tumor initiation in ALDH‐positive breast cells (Kim *et al*., 2013). However, nothing has yet been reported regarding the regulatory effects and mechanisms of HIF‐2α on the drug resistance in OCSCs, which is worthy of further investigation.

Human breast cancer resistance protein (BCRP), also known as ATP‐binding cassette subfamily G member 2 (ABCG2), is a vital member of the ATP‐binding cassette transporter family. BCRP expression is closely correlated with CSC‐like properties; as such, it is also an important CSCs markers (Gilani *et al*., 2012; Szotek *et al*., 2006). BCRP was first identified in the multidrug resistance (MDR) breast cancer cell line MCF‐7/AdrVp in 1998 (Doyle *et al*., 1998). BCRP normally functions to detoxify and protect normal cells from xenobiotics. Increased BCRP expression confers the MDR on CSCs, including OCSCs (Hu *et al*., 2010), by pumping chemotherapeutic agents such as adriamycin (ADR) and mitoxantrone (MX) out of the cell, thereby effectively reducing the intracellular concentrations of these drugs (Natarajan *et al*., 2012). It has been reported that the inhibition of BCRP expression effectively reduces ‘stemness’ and chemotherapy resistance in ovarian cancer cells (Januchowski *et al*., 2016; Jung *et al*., 2015). Reversing the chemotherapy resistance in tumors caused by OCSC via targeting BCRP might lead to the development of new strategies to cure ovarian cancer clinically.

In the present study, we found that HIF‐2α expression was elevated by both hypoxia and ADR treatment, and silencing HIF‐2α increased the sensitivity of the OVCAR‐3 and CAOV‐3 sphere‐forming cells (OVCAR‐3 S and CAOV‐3 S) to ADR. Moreover, we demonstrated that HIF‐2α overexpression enhances drug resistance of OCSCs to ADR through directly promoting *BCRP* transcription and increasing ADR transportation. These findings provide evidence for a novel mechanism by which HIF‐2α regulates the resistance of OCSCs to ADR by directly promoting *BCRP* expression and thereby enhancing ADR transportation.

## 2 Materials and methods

### 2.1 Cell lines and culture

The human ovarian cancer cell lines OVCAR‐3 and CAOV‐3 were obtained in 2015 from the American Type Culture Collection. Cells were maintained in RPMI‐1640 medium (HyClone, Logan, Utah, USA) supplemented with 10% FBS (HyClone), 1% penicillin (100 U·mL^−1^, Invitrogen, Carlsbad, CA, UK), and 1% streptomycin (100 mg·mL^−1^, Invitrogen) at 37 °C and 5% CO_2_. Only cells of passage number < 20 were used for experiments.

### 2.2 Ovarian cancer spheroids culture and formation assay

Spheroids were cultured as previously reported by Wang *et al*. (2012). Briefly, OVCAR‐3 and CAOV‐3 cells, at a density of 1 × 10^5^ cells·mL^−1^, were cultured in suspension in serum‐free DMEM‐F12 medium (HyClone) with 2% B27 (Invitrogen), 20 ng·mL^−1^ EGF (Peprotech, Princeton, NJ, USA), and 10 ng·mL^−1^ bFGF (Peprotech) in ultra‐low adherence dishes (Corning, Corning, NY, USA). Next, cells growing into nonadherent spherical clusters were called OVCAR‐3 spheroids (OVCAR‐3 S) and CAOV‐3 spheroids (CAOV‐3 S).

For exposure to hypoxic conditions, OVCAR‐3 S and CAOV‐3 S cells were incubated at 1% O_2_ for 48 h in a controlled hypoxia incubator (HERACELL 150i, Thermo Fisher Scientific, Waltham, MA, USA) at 37 °C before the indicated analysis. For the analysis of sphere formation, after OVCAR‐3 S and CAOV‐3 S cells were treated with ADR (60 nm) alone, 1% O_2_ alone, or ADR (60 nm) + 1% O_2_ for 48 h, the cells were collected, digested into single cells, and plated in 6‐well ultra‐low adherent plates with 2000 cells·well^−1^ in above sphere formation medium (2 mL). After culture for 7–14 days, the number of the spheres/2000 cells was counted under an inverted microscope (Nikon TE2000‐U, Tokyo, Japan).

### 2.3 Lentivirus transfection

Transfection with short hairpin RNA (shRNA) was performed according to the manufacturer's instructions. For shRNA knockdown analysis, OVCAR‐3 S and CAOV‐3 S cells were transfected with shRNA against *HIF‐1A* (sh‐*HIF‐1A*, 5′‐GGAAGAACTATGAACATAA‐3′) and *EPAS1* (sh‐*EPAS1*, 5′‐GTTCTGGTGACTCTTGGTC‐3′) lentiviral transduction particles (MOI‐10 : 1) in the presence of 5 μg·mL^−1^ polybrene (Shanghai Genechem Co., Ltd, Shanghai, China). For cDNA knockin analysis, OVCAR‐3 and CAOV‐3 cells were transfected with lentiviral transduction particles encoding *HIF‐1A* (*HIF‐1A*‐cDNA) and *EPAS1* (*EPAS1*‐cDNA) (Shanghai Genechem Co., Ltd). Eight hours after transfection, the medium was replaced with fresh medium. Forty‐eight or seventy‐two hours after transduction, GFP was observed under a fluorescence microscope (Nikon TE2000‐U) and cells were collected for the experiments.

### 2.4 Western blotting

Western blot analysis was conducted as previously described (He *et al*., 2015). The primary antibodies were Nanog (1 : 1000, Cell Signaling Technology, Beverly, MA, USA, #4548), OCT4 (1 : 1000, Cell Signaling Technology, #2750), CD133 (1 : 1000, Cell Signaling Technology, #5741), HIF‐1α (1 : 1000, Abcam, Cambridge Science Park, Cambridge, USA, ab39266), HIF‐2α (1 : 1000, Abcam, ab72130), β‐actin (1 : 500, Abcam, ab92611), and BCRP (1 : 800, Abcam, ab26056). The bands were visualized by enhanced chemiluminescence. The band intensities were quantitatively analyzed using imagej Software (National Institutes of Health, Bethesda, MD, USA).

### 2.5 Quantitative reverse transcription PCR (qRT‐PCR)

Total RNA isolation and reverse transcription were performed using methods previously described (Ma *et al*., 2013). The β‐actin gene was used as an internal control. The primer sequences are as follows: β‐actin, forward, 5′‐TCCTCCCTGGAGAAGAGCTA‐3′, reverse, 5′‐TCCTGCTTGCTGATCCACAT‐3′; HIF‐1α, forward, 5′‐ACCATGCCCCAGATTCAGG‐3′, reverse, 5′‐AGTGCTTCCATCGGAAGGACT‐3′; and HIF‐2α, forward, 5′‐CTACGCCACCCAGTACCAGG‐3′, reverse, 5′‐GACACCTTGTGGGCTGACG‐3′. The 2^−∆∆Ct^ method was used to calculate fold changes relative to the control.

### 2.6 Flow cytometry

We utilized flow cytometry in order to compare CD133 expression in OVCAR‐3 vs. OVCAR‐3 S cells and CAOV‐3 vs. CAOV‐3 S cells. Cells were suspended at a density of 1 × 10^6^ cells·mL^−1^ in PBS and incubated with fluorescence isothiocyanate (FITC)‐conjugated antibodies against CD133 (1 : 20, BD Pharmingen, San Diego, CA, USA) for 30 min at 4 °C in darkness. For detection of ALDH activity in OVCAR‐3 vs. OVCAR‐3 S cells and CAOV‐3 vs. CAOV‐3 S cells using the ALDEOFLUOR assay according to the manufacturer's instructions (Stem Cell Technologies, Vancouver, Canada), the ALDH‐positive population was defined as cells with increased FITC fluorescence. The gates were determined using diethylaminobenzaldehyde (DEAB)‐treated cells. Single‐cell suspensions were analyzed by flow cytometry.

An ADR accumulation assay was performed as previously described (Zhao *et al*., 2016). Briefly, OVCAR‐3 S and CAOV‐3 S cells transfected with sh‐*HIF‐2*α or sh‐NC lentiviral transduction particles or OVCAR‐3 and CAOV‐3 cells transfected with *EPAS1*‐cDNA or NC‐cDNA lentiviral transduction particles were incubated with 5 μm ADR at 37 °C for 2 h in darkness. Next, cells were collected and washed with ice‐cold PBS. Intracellular ADR levels were determined by measuring mean fluorescence intensity (MFI) using a flow cytometer (Becton‐Dickinson, Franklin Lakes, NJ, USA).

### 2.7 Soft agar colony formation assay

The soft agar colony formation assay was performed as described previously (Mullendore *et al*., 2009). In brief, 1.2% SeaPlaque low melting temperature agarose (Lonza Rockland, ME, Thomaston, USA) in phenol red‐free medium supplemented with 20% FBS was coated in 6‐well plates as a bottom layer. 2000 OVCAR‐3 or CAOV‐3 cells were mixed in 0.6% agarose and the same medium as a middle layer, and a top layer was 600 μL medium only. The colonies were stained with 100 μL MTT (5 mg·mL^−1^) after incubating at 37 °C in 5% CO_2_ for 3 weeks. Colonies were counted using the analysis software quantity one (Bio‐Rad, Hercules, CA, USA).

### 2.8 Cell counting kit (CCK‐8) assay

Cell viability was determined using a CCK‐8 kit (Dojindo, Gaithersburg, MD, USA). Cells (5000 cells·well^−1^) were plated in 96‐well ultra‐low adhesion plates. To determine IC_50_ values, OVCAR‐3 vs. OVCAR‐3 S and CAOV‐3 vs. CAOV‐3 S cells were treated with different concentrations of ADR (Sigma), MX (Sigma, Darmstadt, Hessen, Germany), paclitaxel (PTX, Sigma), etoposide (VP‐16, Sigma), or cisplatin (DDP, Sigma) for 48 h. Next, the cells in each well were incubated with 10 μL WST‐8 for 4 h at 37 °C. The absorbance was then measured at 450 nm using an Anthos 2010 microplate reader (Anthos Labtec Instruments GmbH, Wals, Austria).

### 2.9 Transwell invasion assays

Transwell invasion assays were performed as previously described (Fu *et al*., 2016).

### 2.10 Dual‐luciferase reporter assay

293T or OVCAR‐3 cells (1 × 10^4^ per well) were seeded in triplicate in 96‐well plates. After 24 h, cells were transiently transfected with either 0.1 μg GV238‐basic vectors containing upstream promoter regions of *BCRP* (GV238‐BCRP‐WT), vectors containing mutated HRE sequence binding sites (GV238‐*BCRP*‐mut), or NC promoter plasmids and 0.01 μg Renilla luciferase plasmid and 0.2 μg *EPAS1*‐cDNA or NC‐cDNA plasmids (all from Shanghai Genechem Co., Ltd) using Lipofectamine 3000 transfection regent. Luciferase and Renilla signals were measured 48 h after transfection using the Dual Luciferase Reporter Assay Kit (Promega, Madison, WI, USA) according to the manufacturer's protocol.

### 2.11 ChIP assay

ChIP assays were performed using a ChIP assay kit (Beyotime, Haimen, China) following the manufacturer's instructions. Briefly, OVCAR‐3 or OVCAR‐3 S cells were cultured under hypoxic conditions (1% O_2_) for 48 h in a controlled hypoxia incubator at 37 °C in a 90‐mm culture dish. Next, cells were resuspended in SDS lysis buffer for sonication and further immunoprecipitation with anti‐HIF‐2α antibody (1 : 100, abcam). Normal rabbit IgG was used as a negative control. Immune complexes were then collected, washed, and eluted. The presence of HIF‐2α on the promoter region was analyzed by amplifying the DNA fragment (199 bp) corresponding to upstream HRE motifs in the *BCRP* gene using the following set of primers (forward: 5′‐AATGAGCGCCTGGTGATTCT‐3′, and reverse: 5′‐CGATAAGCGCCCTGCGA‐3′) in the PCR product.

### 2.12 *In vivo* xenograft experiments

Female BALB/c (nu/nu) mice (Hua Fukang Biological Technologies Inc, Beijing, China), 6–8 weeks of age, were bred in pathogen‐free conditions at the Animal Center of China Medical University. The Animal Research Committee at China Medical University approved all animal studies.

To study the tumorigenic ability of OVCAR‐3 and OVCAR‐3 S cells, equal numbers (1 × 10^6^) of cells were suspended in 200 μL PBS and Matrigel (1 : 1, BD Biosciences, Franklin Lakes, NJ, USA) and subcutaneously inoculated into the right flank of nude mice (*n *=* *6 per group). Tumor volume (V) = length × width^2^/2. Mice were euthanized 35 days after the initial injection of the cells, and then, xenograft tumors were excised for the study.

To study the effects of silencing HIF‐2α on the response of the xenograft tumors to ADR, 1 × 10^6^ OVCAR‐3 S cells stably transfected with sh‐*EPAS1* lentiviral transduction particles or sh‐NC as a control were subcutaneously inoculated into nude mice as described above. Thirteen days after inoculation, the transplanted nude mice were randomly divided into four groups: sh‐NC alone, sh‐NC + ADR, sh‐*EPAS1* alone, sh‐*EPAS1*+ ADR groups (*n *=* *5 per group). The mice in the sh‐NC + ADR and sh‐*EPAS1*+ ADR groups were intraperitoneally injected with ADR (1.5 mg·kg^−1^), while the mice in the sh‐NC and sh‐*HIF‐2*α groups were intraperitoneally injected with DMSO once every other day. Tumor volume was calculated and recorded. Mice were sacrificed 4 weeks after the initial injection, and tumors were weighted and harvested for further study.

### 2.13 High‐performance liquid chromatography–tandem mass spectrometry (HPLC‐MS/MS)

The procedure was carried out according to methods with the modifications (Ma *et al*., 2015). For the preparation of the tumor tissues, xenograft tumors from the sh‐NC + ADR and sh‐*EPAS1*+ ADR groups were weighed, gradually added to 1.5 mL of 0.9% sodium chloride solution, and homogenized. For preparation of transfected cells, cells were cultured in 6‐well plates (3 × 10^5^/well). Twenty‐four hours after transfecting OVCAR‐3 S and CAOV‐3 S cells with sh‐*EPAS1* lentiviral vector particles and OVCAR‐3 and CAOV‐3 cells with *EPAS1*‐cDNA lentiviral vector particles, ADR was added to cells at the final concentration of 8 μm for 2 h. After washing cells three times with PBS, cells were cultured in fresh medium without ADR at 37 °C for 1 h in darkness. Next, cells were lysed by repeated cycles of freezing and thawing and centrifuged at 13 680 ***g*** for 5 min at 4 °C. One hundred microliters of the supernatant was mixed with 10 μL of pioglitazone solution as an internal standard (IS) at a final concentration of 1 μg·mL^−1^, mixed with 50 μL methanol water (1 : 1), and then vortexed for 30 S. The mixtures were shaken for 2 min after the addition of another 250 μL methanol. After centrifugation at 18 630 ***g*** for 10 min at 4 °C, the supernatant was separated and transferred to an auto‐sample vial and an aliquot of 5 μL was used for HPLC‐MS analysis. Three independent experiments of each sample were analyzed. The biological samples were analyzed with an Agilent series 1290 UHPLC system (Agilent Technologies, Santa Clara, CA, USA) coupled to an AB 3500 triple‐quadrupole mass spectrometer (Agilent Technologies) via an electrospray ionization (ESI) interface. All acquisition data were analyzed using the analyst software version 1.6.3 package (Agilent Technologies).

### 2.14 Patients

Ovarian cancer tissues were obtained from 115 ovarian cancer patients who underwent surgery at Shengjing Hospital of China Medical University, Liaoning Province, China, between 2010 and 2012. The study has been approved by the Institutional Review Board of China Medical University, and all subjects gave their informed consent prior to their inclusion in the study.

### 2.15 Immunohistochemistry

Immunohistochemistry staining was performed as previously described (He *et al*., 2015). Briefly, sections from paraffin‐embedded tumor tissues from ovarian cancer patients or transplanted nude mice were incubated with primary antibodies against HIF‐2α (1 : 100), HIF‐1α (1 : 100), CD133 (1 : 100), aldehyde dehydrogenase isoform 1A1 (ALDH1A1; 1 : 200), and BCRP (1 : 100). The immunoreactivity for ovarian cancer patient sections was evaluated by multiplying the intensity score with the score for the percentage of positively stained cells. Five random fields without overlaps from each section were counted. The intensity scores were as follows: 0 for no staining, 1 for weak staining, 2 for moderate staining, and 3 for strong staining. The scores for the percentage of positively stained cells ranged between 0% and 100%. The integrated optical density (IOD) of the immunoreactivity of transplanted nude mice sections was analyzed using image‐pro plus 6.0 software (Georgia Avenue Silver Spring, MD, USA).

### 2.16 Statistical analysis

Data were analyzed using graphpad prism 7 (GraphPad Software Inc., San Diego, CA, USA). Quantitative data are presented as the mean ± standard deviation (SD) of at least three experiments. A Student's *t*‐test was used to compare the differences between two groups. One‐way analysis of variance (anova) was used for multiple comparisons. *P *<* *0.05 was considered statistically significant.

## 3 Results

### 3.1 Ovarian cancer sphere‐forming cells with OCSC‐like characteristics are resistant to chemotherapeutic drugs and overexpress the CSC marker, BCRP

**Figure 1:**
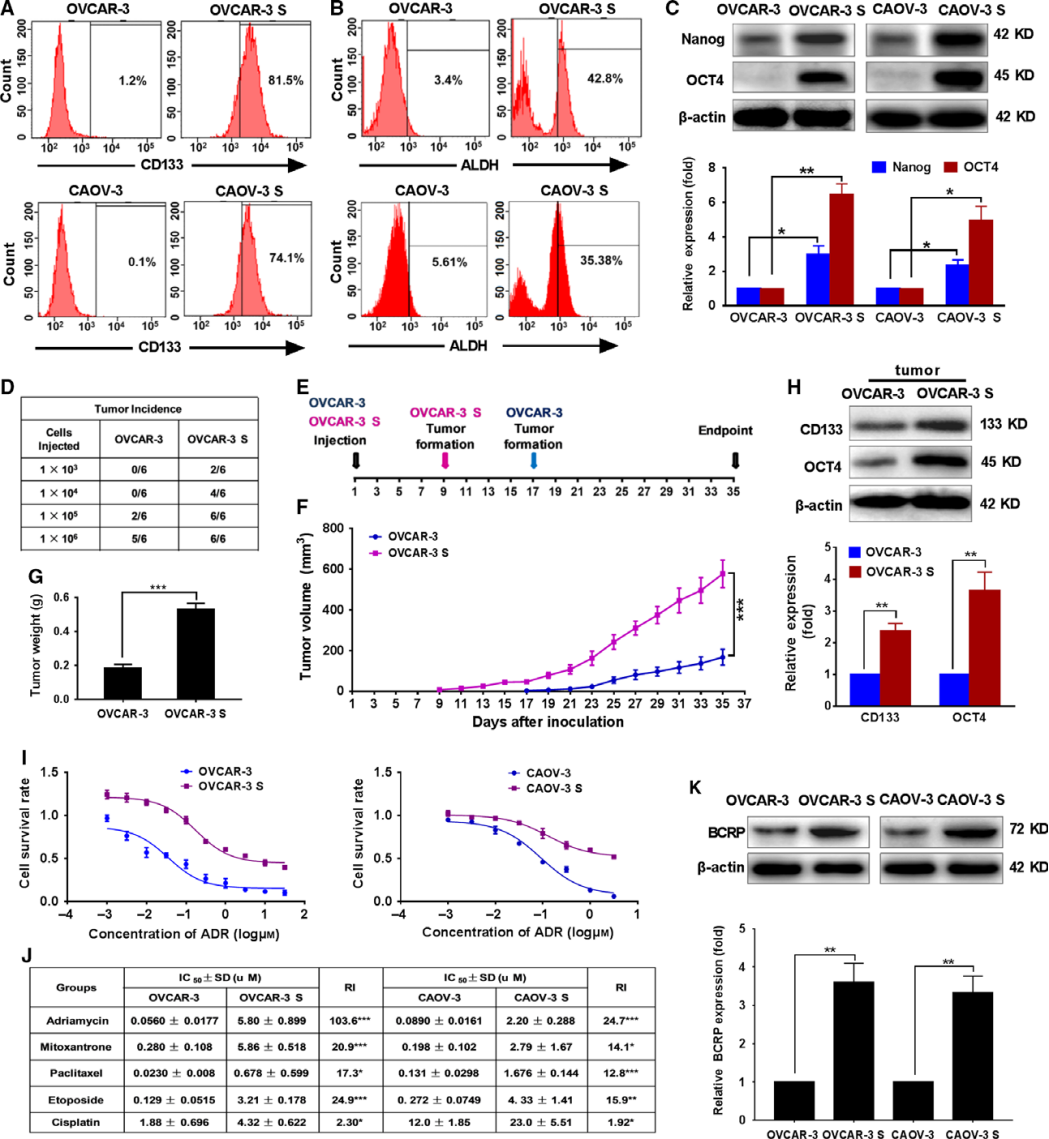
OVCAR‐3 S and CAOV‐3 S cells possess OCSC‐like properties, are resistant to ADR, and overexpress the CSC marker, BCRP. (A) Representative images indicating the percentage of CD133‐positive cells in OVCAR‐3 vs. OVCAR‐3 S cells and CAOV‐3 vs. CAOV‐3 S cells as determined by flow cytometry. (B) Representative images of the percentage of ALDH‐positive cells in OVCAR‐3 vs. OVCAR‐3 S cells and CAOV‐3 vs. CAOV‐3 S cells by flow cytometry. (C) The expression levels of Nanog and OCT4 were measured by western blot analysis. β‐actin was used as a loading control. The expression level of Nanog or OCT4 was normalized to that of β‐actin. The Nanog or OCT4 expression level in OVCAR‐3 or CAOV‐3 cells was set as 1. Data are presented as mean ± SD from three independent experiments. *P <* *0.05, **P <* *0.01. (D) Tumor incidence in female BALB/c athymic nude mice at 5 weeks after subcutaneous injection with the indicated number of OVCAR‐3 and OVCAR‐3 S cells *in vivo* (*n *=* *6 per group). (E) A diagram showing the time of tumor formation in the BALB/c mice transplanted with the OVCAR‐3 S cells or OVCAR‐3 cells (1 × 10^6^). (F) Tumor volumes were calculated as described in the Methods (*n *=* *5 per group). ***P <* *0.001. (G) The weight of tumors induced by inoculation of OVCAR‐3 and OVCAR‐3 S cells after mice were sacrificed 35 days after cell inoculation (*n *=* *5 per group). (H) The expression of CD133 and OCT4 in tumors transplanted with OVCAR‐3 S cells or OVCAR‐3 cells was determined by western blot analysis. β‐actin was used as a loading control. The expression level of CD133 or OCT4 was normalized to that of β‐actin. The CD133 or OCT4 expression level in OVCAR‐3 cells was set as 1. Data are presented as mean ± SD from three independent experiments. **P <* *0.01. (I) Cell survival rate was analyzed by CCK‐8 assays 48 h after OVCAR‐3/OVCAR‐3 S and CAOV‐3/CAOV‐3 S cells were treated with different concentrations of ADR. (J) The comparison of IC_50_ and resistant index (RI) values of ADR, MX, PTX, VP‐16, and DDP in OVCAR‐3 vs. OVCAR‐3 S cells and CAOV‐3 vs. CAOV‐3 S cells. Data are presented as mean ± SD from three independent experiments. *P <* *0.05, **P <* *0.01, ***P <* *0.001. (K) The protein expression levels of BCRP were analyzed by western blot in OVCAR‐3 S and CAOV‐3 S cells versus their parental cells. β‐actin was used as a loading control. The expression level of BCRP was normalized to that of β‐actin. The BCRP expression level in OVCAR‐3 cells or CAOV‐3 cells was set as 1. Data are presented as mean ± SD from three independent experiments. **P <* *0.01. Statistical significance was evaluated by Student's *t*‐test.

Serum‐free suspension culture is a common and reliable method for the culture of ovarian cancer stem cells (OCSCs). We isolated the sphere‐forming cells (OVCAR‐3 S and CAOV‐3 S) from OVCAR‐3 and CAOV‐3 ovarian cancer cell lines by culturing cells in CSC culture medium according the methods described by Vermeersch *et al*. (2015) and Wang *et al*. (2012). Both CD133 expression and ALDH activity were higher in OVCAR‐3 S and CAOV‐3 S cells than their parental cells, as determined using flow cytometry (Figs. 1A,B and S1A). The expression levels of Nanog and OCT4, stem cell markers, were significantly increased in OVCAR‐3 S and CAOV‐3 S cells via immunoblot analysis (Fig. 1C). Moreover, we found that OVCAR‐3 S and CAOV‐3 S cells formed more colonies than OVCAR‐3 and CAOV‐3 cells in a soft agar colony formation assay (Fig. S1B). Transwell invasion assays showed that the number of OVCAR‐3 S and CAOV‐3 S cells that invaded into the lower transwell chamber was significantly greater than the number parental cells that were able to invade (Fig. S1C). We also found that OVCAR‐3 S and CAOV‐3 S cells re‐differentiated into their parental OVCAR‐3 and CAOV‐3 cells in DMEM culture medium supplemented with 10% FBS for 72 h (Fig. S1D) by referring to Ponti *et al*.'s (2005) and Wu *et al*.'s (2010) reports. In addition, we observed that injection of OVCAR‐3 S cells (1 × 10^3^) could form tumors in xenograft mice, whereas 1 × 10^5^ parental OVCAR‐3 cells were required to generate tumors *in vivo* (Fig. 1D). Five weeks after 1 × 10^6^ OVCAR‐3 S cells were injected, the average tumor volume and weight of the resulting xenograft tumors were much higher than those of parental OVCAR‐3 xenograft tumors (Fig. 1E–G). The expression levels of CD133 and OCT4 proteins were also higher in OVCAR‐3 S xenograft tumors (Fig. 1H). These data suggest that OVCAR‐3 S and CAOV‐3 S cells obtained from serum‐free suspension culture possess ovarian CSC‐like properties such as self‐renewal, strong invasion capacity, differentiation potential, and high tumorigenicity.

Chemoresistance is one of the most important characteristics of OCSCs. We assessed the sensitivity difference of OVCAR‐3 vs. OVCAR‐3 S and CAOV‐3 vs. CAOV‐3 S cells to ADR, MX, PTX, VP‐16, and DDP treatment for 48 h using CCK‐8 assays. We found that OVCAR‐3 S and CAOV‐3 S cells exhibited resistance to all these drugs with higher IC_50_ values as compared with their parental cells (Figs 1I,J and S2A–D). OVCAR‐3 S and CAOV‐3 S cells were most strongly resistant to ADR; the resistant indices were 103.6 and 24.7 relative to OVCAR‐3 and CAOV‐3 cells, respectively. Thus, we utilized ADR to evaluate the mechanisms of OCSC resistance in subsequent experiments.

Breast cancer resistance protein, as a major CSC marker, plays an important role in the MDR of CSCs. To evaluate whether BCRP confers ADR resistance on OVCAR‐3 S and CAOV‐3 S cells, we measured the BCRP protein expression levels in OVCAR‐3 S and CAOV‐3 S as compared to their parental cells. Western blot analysis showed that the expression levels of BCRP in OVCAR‐3 S and CAOV‐3 S cells were 3.61‐fold and 3.35‐fold higher than in OVCAR‐3 and CAOV‐3 parental cells, respectively (Fig. 1K). These data suggest BCRP may contribute to ADR resistance in OCSCs.

### 3.2 Hypoxia mediates the resistance of OCSCs to ADR by inducing high expression of HIF‐2α

**Figure 2:**
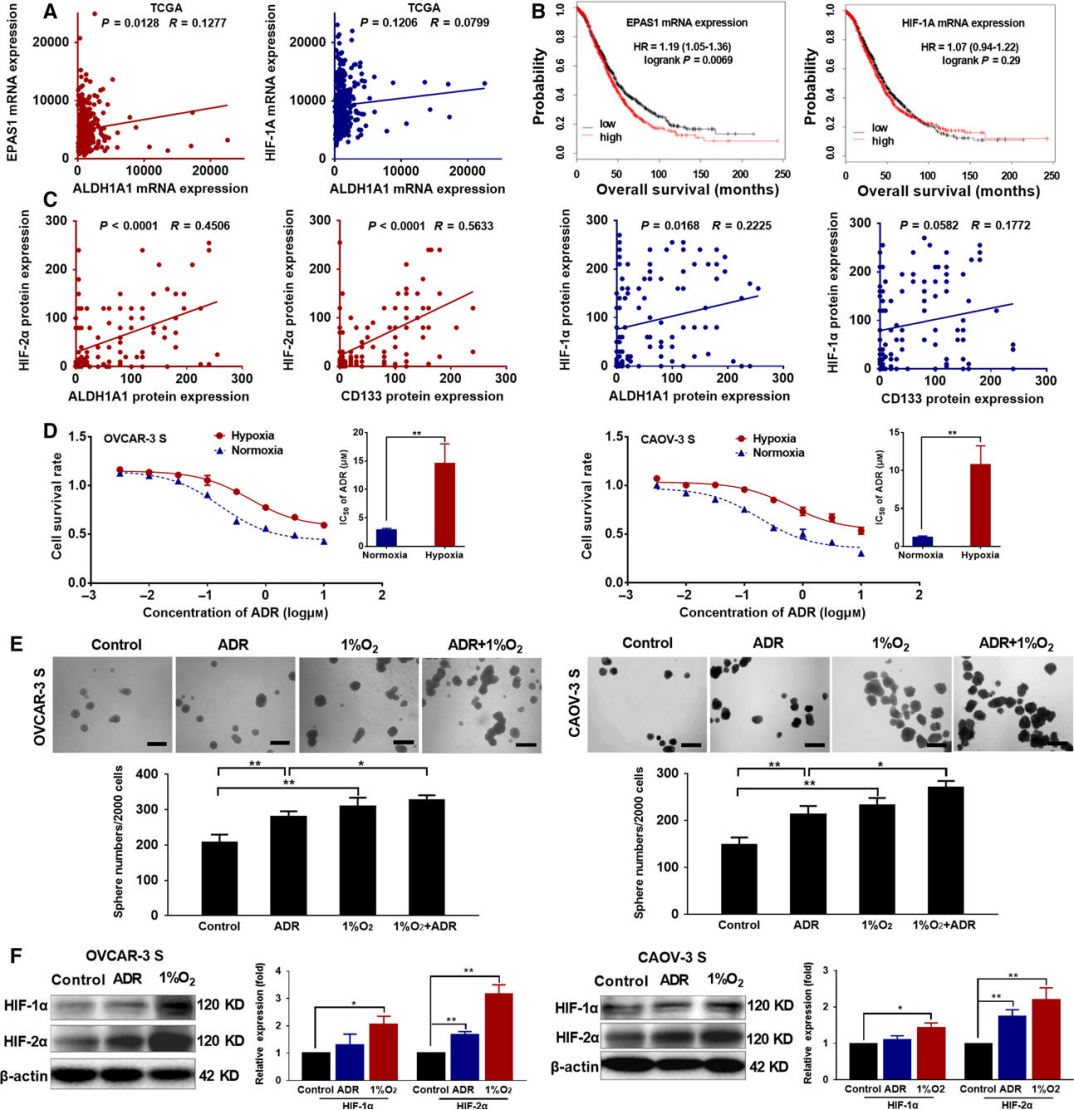
Hypoxia increases the resistance of OCSCs to ADR. (A) The relationship between mRNA expression of HIF‐1A or EPAS1 and ALDH1A1 was analyzed in 379 ovarian cancer cases from TCGA data. (B) The relationship between mRNA expression of HIF‐1A or EPAS1 and the outcomes of 1657 ovarian cancer cases was analyzed using the online tool, KM plotter (http://www.kmplot.com). (C) The protein expression correlation between HIF‐1α or HIF‐2α and ALDH1A1 or CD133 in 115 ovarian tumor tissues was analyzed using Pearson correlation analysis. (D) Cell survival rate was analyzed by CCK‐8 assays, and the IC_50_ values were compared 48 h after OVCAR‐3 S and CAOV‐3 S cells were treated with different concentrations of ADR under normoxic or hypoxic conditions (1% O_2_). Data are presented as the mean ± SD from three independent experiments. **P <* *0.01, compared with the cells under normoxia. (E) Sphere formation assays were carried out in OVCAR‐3 S and CAOV‐3 S cells 48 h after treatment with ADR (60 nm) alone, 1% O_2_ alone, or ADR (60 nm) + 1% O_2_. The length of the scale bars is 200 μm. Data are presented as the mean ± SD from three independent experiments. *P <* *0.05, **P <* *0.01. (F) Protein expression levels of HIF‐1α and HIF‐2α were analyzed by western blot in OVCAR‐3 S and CAOV‐3 S cells 48 h after treatment with 60 nm ADR or hypoxia (1% O_2_). β‐actin was used as a loading control. The expression levels of HIF‐1α or HIF‐2α were normalized to that of β‐actin. The HIF‐1α or HIF‐2α expression levels in OVCAR‐3 cells or CAOV‐3 cells without ADR or exposure to hypoxia (1%O_2_) were set as 1. Data are presented as the mean ± SD from three independent experiments. *P <* *0.05, **P <* *0.01. Statistical significance was evaluated by one‐way anova.

Hypoxia can promote ‘stemness’ maintenance of CSCs and is a functional component of CSC niches (Takakura, 2012). In order to evaluate the association HIF‐1α or HIF‐2α expression with stem cells markers in clinical ovarian cancer patients, we first used the Cancer Genome Atlas (TCGA) database to analyze the relationship between mRNA expression of *HIF‐1A* or *EPAS1* and ALDH1A1, a CSC marker, in 379 ovarian cancer cases. The mRNA expression of *EPAS1* (but not *HIF‐1A*) showed a significantly positive correlation with mRNA expression of ALDH1A1 (Fig. 2A). Next, we used the online tool, KM plotter (http://www.kmplot.com), to analyze the relationship between mRNA expression of HIF‐1α or HIF‐2α and the outcomes of 1657 ovarian cancer cases. We found that higher mRNA expression of *EPAS1* was significantly positively associated with shorter OS of patients, but higher mRNA expression of *HIF‐1A* had no positive association with worse outcomes of patients (Fig. 2B). In addition, we tested the protein expression of HIF‐1α or HIF‐2α and ALDH1A1 or CD133, another CSC marker, in 115 ovarian tumor tissues using immunohistochemical staining. The clinicopathological features were shown in Table S1. A Pearson correlation analysis indicated that expression of HIF‐2α was significantly positively correlated with the expression of ALDH1A1 and CD133, but the expression of HIF‐1α was only positively correlated with the expression of ALDHA1 (Figs 2C and S3). These data suggest HIF‐2α may contribute to the ‘stemness’ of OCSCs and poor outcomes of ovarian cancer patients.

To study the effects of hypoxia on the sensitivity of OCSCs to ADR, we analyzed the cell survival rate via CCK‐8 assay after culturing OVCAR‐3 S and CAOV‐3 S cells with different concentrations of ADR under hypoxic conditions (1% O_2_) for 48 h. We found that hypoxia decreased the response of OVCAR‐3 S and CAOV‐3 S cells to ADR; ADR IC_50_ values were higher than when cells were grown under normoxia (Fig. 2D). In addition, sphere formation assays demonstrated that either ADR treatment (60 nm) alone or hypoxia treatment alone increased sphere numbers of OVCAR‐3 S and CAOV‐3 S cells as compared to untreated cells. Also, compared with cells treated with ADR under normoxic conditions, OVCAR‐3 S and CAOV‐3 S cells treated with ADR under hypoxic conditions formed more spheres (Fig. 2E), suggesting that hypoxia can induce the resistance of OCSCs to ADR.

To study whether HIF‐1α or HIF‐2α plays a role in the OCSC resistance to ADR induced by hypoxia, we measured protein expression levels of HIF‐1α and HIF‐2α in OVCAR‐3 S and CAOV‐3 S cells 48 h after hypoxic treatment (1% O_2_) or ADR (60 nm) by western blot. We found that hypoxia induced higher expression of HIF‐1α and especially HIF‐2α in OVCAR‐3 S and CAOV‐3 S cells as compared to untreated cells. Importantly, we observed that treatment with ADR significantly upregulated HIF‐2α expression, but not HIF‐1α expression in OVCAR‐3 S and CAOV‐3 S cells (Fig. 2F). We also found the increased HIF‐1α, especially HIF‐2α expression in OVCAR‐3 S and CAOV‐3 S cells relative to their parental cells (Fig. S4A). Additionally, hypoxia culture for 48 h induced the increased HIF‐1α, especially HIF‐2α expression in OVCAR‐3 and CAOV‐3 cells (Fig. S4B).

Collectively, these findings suggest that hypoxia may mediate the resistance of OCSCs to ADR by inducing high expression of HIF‐2α.

### 3.3 HIF‐2α not HIF‐1α mediates the resistance of OCSCs to ADR

To further assess whether HIF‐1α or HIF‐2α contributes to ADR resistance induced by hypoxia in OCSCs, we first transduced OVCAR‐3 S and CAOV‐3 S cells with lentiviral vectors carrying promoters driving GFP and shRNA against *HIF‐1A* (sh‐*HIF‐1A*), *EPAS1* (sh‐*EPAS1*), or a negative control (sh‐NC) to deplete HIF‐1α or HIF‐2α. We also transduced OVCAR‐3 and CAOV‐3 cells with lentiviral vectors containing cDNA for *HIF‐1A* (*HIF‐1A*‐cDNA), *EPAS1* (*EPAS1*‐cDNA), or a negative control (NC‐cDNA) resulting in overexpression of HIF‐1α or HIF‐2α. Approximately 80% transduction efficiency was observed by examining GFP expression under a fluorescence microscope (Fig. S5A). A significant reduction in mRNA (Fig. S5B) and protein expression level (Fig. S5C) of HIF‐1α or HIF‐2α was observed in OVCAR‐3 S and CAOV‐3 S cells transduced with sh‐*HIF‐1A* or sh‐*EPAS1* lentivirus relative to controls. Both the high transduction efficiency (Fig. S6A) and the increased mRNA (Fig. S6B) and protein expression (Fig. S6C) of HIF‐1α and HIF‐2α in *HIF‐1A*‐cDNA‐transduced and *EPAS1*‐cDNA‐transduced cells indicate that ovarian cancer cells successfully overexpressed either HIF‐1α or HIF‐2α.

**Figure 3:**
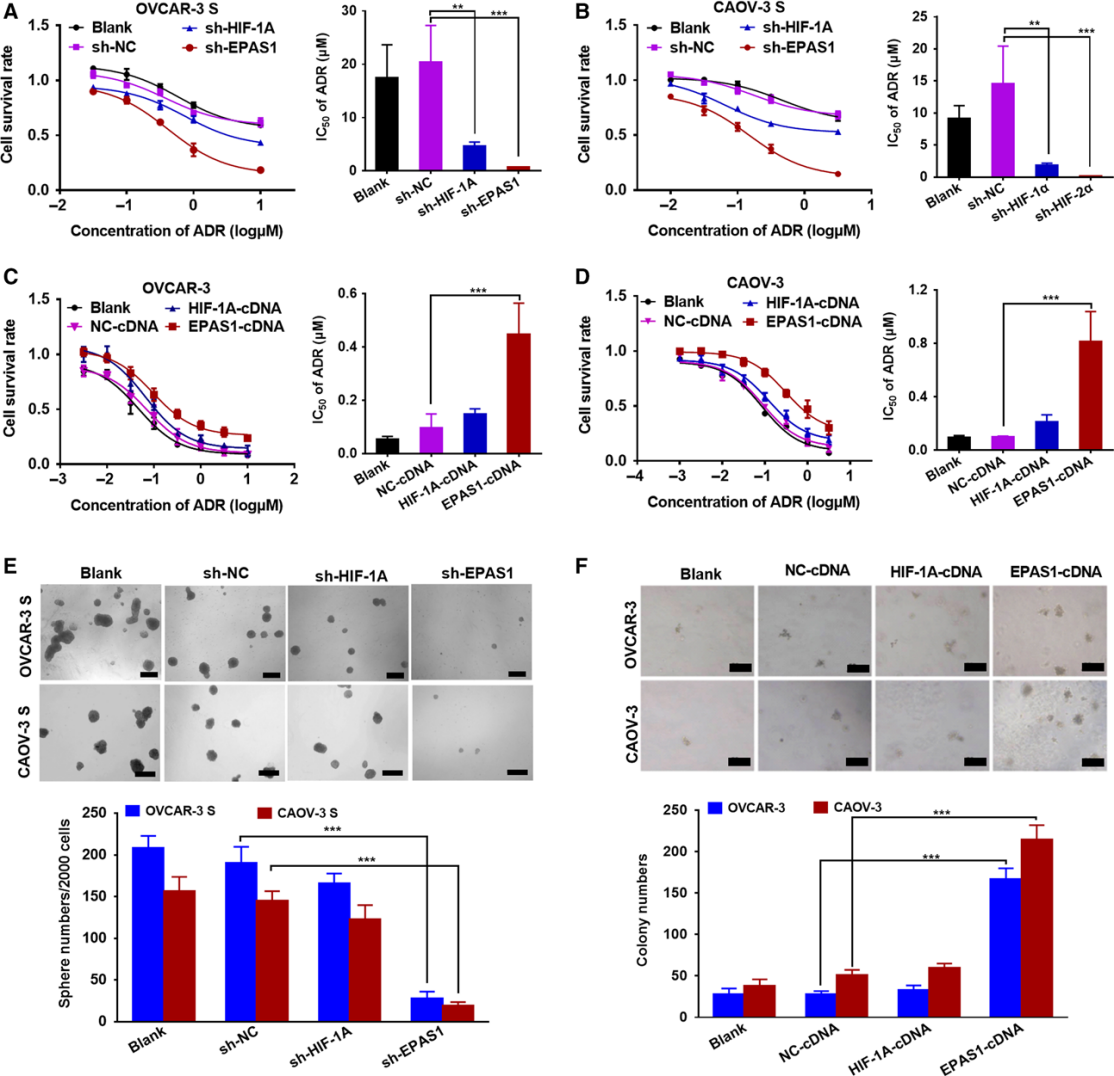
The influences altered expression levels of HIF‐2α on the sensitivity of OCSCs to ADR. Cell survival rate was analyzed by CCK‐8 assays and the comparison of IC_50_ values 48 h after (A) OVCAR‐3 S and (B) CAOV‐3 S cells transduced with sh‐*HIF‐1A*, sh‐*EPAS1*, or negative control (sh‐NC) lentivirus and treated with different concentrations of ADR under hypoxic conditions (1% O_2_) for 48 h. Data are presented as the mean ± SD from three independent experiments. **P <* *0.01, ***P <* *0.001. Cell survival rate was analyzed by MTT assays and the comparison of IC_50_ values 48 h after (C) OVCAR‐3 and (D) CAOV‐3 cells were transduced with *HIF‐1A*‐cDNA,*EPAS1*‐cDNA, or NC‐cDNA lentivirus and treated with different concentrations of ADR. Data are presented as the mean ± SD from three independent experiments. ***P <* *0.001. (E) Sphere formation assays were carried out in OVCAR‐3 S and CAOV‐3 S cells transduced with sh‐*HIF‐1A,* sh‐*EPAS1*, or sh‐NC lentivirus. The length of the scale bars is 200 μm. (F) Soft agar colony formation assays were done in the OVCAR‐3 and CAOV‐3 cells transduced with *HIF‐1A*‐cDNA,*EPAS1*‐cDNA, or NC‐cDNA lentivirus. The length of the scale bars is 50 μm. Data are presented as the mean ± SD from three independent experiments. *** *P *<* *0.001. Statistical significance was evaluated by Student's *t*‐test.

A CCK‐8 assay demonstrated that *HIF‐2*α silencing in OVCAR‐3 S and CAOV‐3 S cells dramatically reduced the cell survival rate after treatment of different concentrations of ADR for 48 h as indicated by significantly reduced IC_50_ values relative to the sh‐NC‐transduced cells (Fig. 3A,B). Though *HIF‐1A* silencing also decreased the cell survival rate, the effects were not as pronounced as *EPAS1* silencing. Meanwhile, we found that HIF‐2α, but not HIF‐1α overexpression, resulted in an elevated cell survival rate after treatment with ADR as indicated by increased IC_50_ values in OVCAR‐3 cells and CAOV‐3 cells (Fig. 3C,D). Furthermore, sphere formation assays indicated that *EPAS1* suppression significantly reduced sphere numbers of OVCAR‐3 S and CAOV‐3 S cells, whereas *HIF‐1A* suppression did not (Fig. 3E). In addition, overexpression of HIF‐2α, but not HIF‐1α, significantly increased the sphere numbers of OVCAR‐3 and CAOV‐3 cells, relative to the NC‐cDNA‐transduced cells in colony formation assays (Fig. 3F). Western blot analysis also showed that overexpression of HIF‐2α, but not HIF‐1α, notably increased the protein expression of OCT4 in OVCAR‐3 and CAOV‐3 cells (Fig. S7). Thus, these findings suggest that HIF‐2α, as opposed to HIF‐1α, plays an important role in regulating the resistance of OCSCs induced by hypoxia to ADR.

### 3.4 HIF‐2α regulates the resistance of OCSCs to ADR by affecting the expression and transport function of BCRP

**Figure 4:**
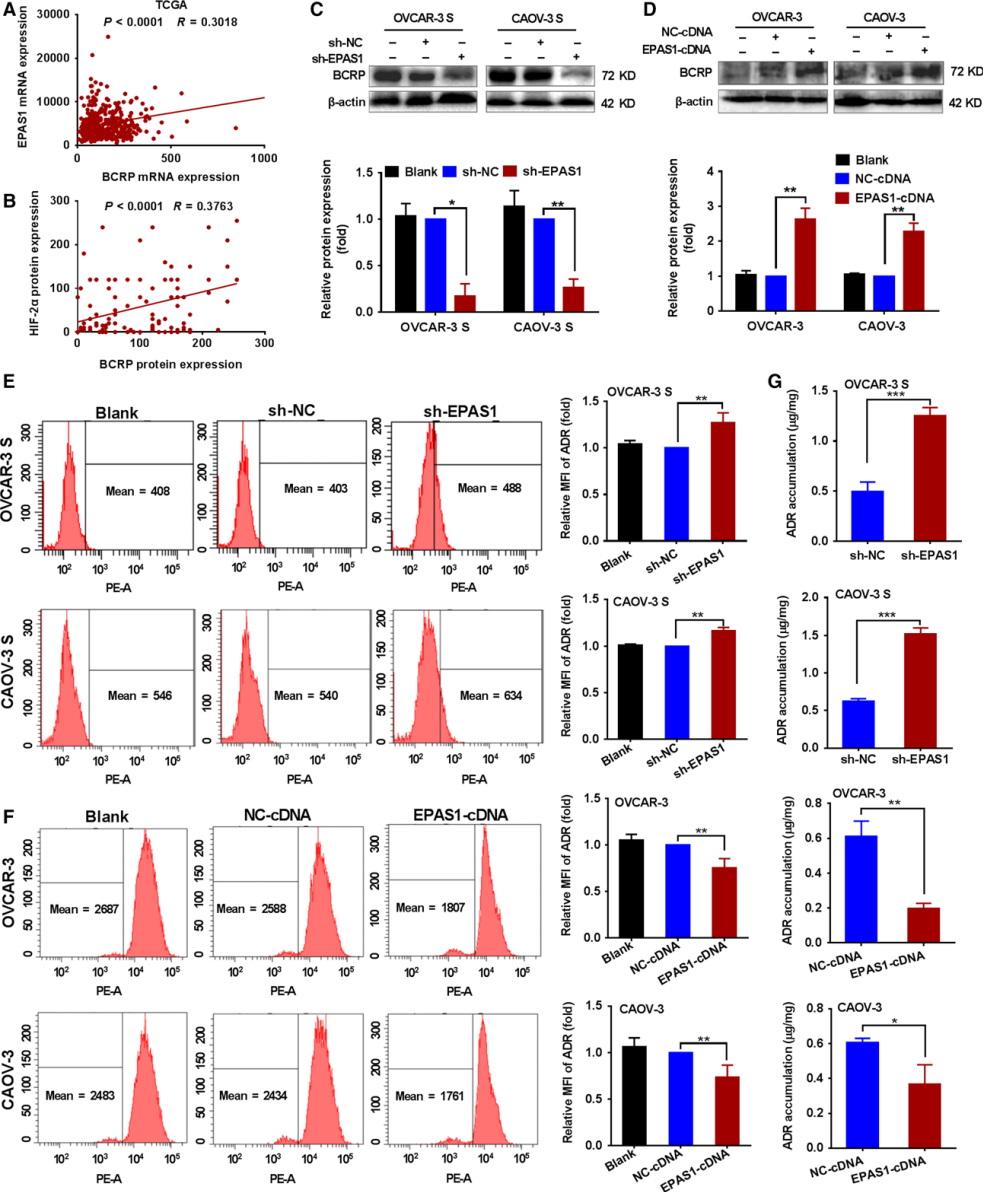
The effects of HIF‐2α on the expression of the BCRP protein and the transport function of BCRP for ADR in OCSCs. (A) TCGA data were used to analyze the relationship between mRNA expression of *EPAS1* and *BCRP* in 379 ovarian cancer cases by linear regression analysis. (B) Correlation between protein expression of HIF‐2α and BCRP in 115 ovarian cancer tissues was analyzed by linear regression analysis. (C) The protein expression of BCRP was analyzed by western blot in OVCAR‐3 S and CAOV‐3 S cells transduced with sh‐*EPAS1* or sh‐NC lentivirus under hypoxic conditions (1% O_2_) for 48 h and (D) in the OVCAR‐3 and CAOV‐3 cells transduced with *EPAS1*‐cDNA or NC‐cDNA lentivirus. β‐actin was a used an endogenous control. BCRP expression levels in the OVCAR‐3 S cells and CAOV‐3 S cells transduced with sh‐NC lentivirus and OVCAR‐3 cells and CAOV‐3 cells transduced with NC‐cDNA were set as 1. Data are presented as the mean ± SD from three independent experiments. *P <* *0.05, **P <* *0.01. (E) Changes in the intracellular accumulation of ADR were determined by flow cytometric analysis in OVCAR‐3 S and CAOV‐3 S cells transduced with sh‐ *EPAS1* or sh‐NC lentivirus under hypoxic conditions (1% O_2_) for 48 h, and (F) in the OVCAR‐3 cells or CAOV‐3 cells transduced with *EPAS1*‐cDNA or NC‐cDNA lentivirus. The MFI of the intracellular accumulation of ADR in OVCAR‐3 S cells or CAOV‐3 S cells transduced with sh‐NC lentivirus and in OVCAR‐3 cells or CAOV‐3 cells transduced with NC‐cDNA was set as 1. Data are presented as mean ± SD from three independent experiments. **P <* *0.01. (G) Changes in intracellular accumulation of ADR in *EPAS1‐*silenced OVCAR‐3 S and CAOV‐3 S cells and (H) *EPAS1‐*overexpressing OVCAR‐3 and CAOV‐3 cells were analyzed by mass spectrometry. Data are presented as the mean ± SD from three independent experiments. *P <* *0.05, **P <* *0.01, ***P <* *0.001. Statistical significance was evaluated by Student's *t*‐test.

How does HIF‐2α regulate the resistance of OCSCs to ADR? Considering the expression of BCRP, a CSC marker, was upregulated in OCSCs, we considered whether the high expression of BCRP contributed to the regulation of the HIF‐2α and thus the resistance of OCSC to ADR. To test this hypothesis, we first used the TCGA data to analyze the relationship between HIF‐2α mRNA expression and BCRP in 379 ovarian cancer cases. The results showed that the HIF‐2α mRNA expression was significantly positively associated with *EPAS1* mRNA expression (Fig. 4A). We also analyzed the correlation between protein expression of HIF‐2α and BCRP in 115 ovarian cancer tissues from our case bank using linear regression analysis. Protein expression of HIF‐2α was also positively correlated with protein expression of BCRP (Fig. 4B).

To evaluate whether HIF‐2α regulates the resistance of OCSCs to ADR by affecting the expression and transport function of BCRP, we first analyzed BCRP protein expression in the OVCAR‐3 S and CAOV‐3 S cells transduced with sh‐*EPAS1* lentivirus or *EPAS1*‐cDNA lentivirus by western blot. HIF‐2α depletion significantly reduced the BCRP protein levels in the OVCAR‐3 S and CAOV‐3 S cells (Fig. 4C), while *EPAS1* overexpression significantly upregulated the protein expression of BCRP in OVCAR‐3 and CAOV‐3 cells (Fig. 4D).

Next, flow cytometric analysis was performed to measure changes in the intracellular accumulation of ADR in *EPAS1‐*silenced OVCAR‐3 S and CAOV‐3 S cells and *EPAS1‐*overexpressing OVCAR‐3 and CAOV‐3 cells. The MFI of ADR in *EPAS1‐*silenced OVCAR‐3 S and CAOV‐3 S cells was increased relative to control cells (Fig. 4E). Conversely, the MFI of ADR in *EPAS1‐*overexpressing OVCAR‐3 and CAOV‐3 cells was reduced compared to control cells (Fig. 4F). We also used mass spectrometry to analyze the changes in intracellular accumulation of ADR in the *EPAS1‐*silenced OVCAR‐3 S and CAOV‐3 S cells and in *EPAS1‐*overexpressing OVCAR‐3 and CAOV‐3 cells. Consistent with the results from flow cytometric analysis, mass spectrometry also showed that silencing *EPAS1* led to increased cellular accumulation of ADR in the OVCAR‐3 S and CAOV‐3 S cells (Figs 4G and S8A–C), while overexpressing HIF‐2α resulted in decreased cellular accumulation of ADR in the OVCAR‐3 and CAOV‐3 cells (Figs 4H and S8D). Therefore, these findings indicate that HIF‐2α might regulate the resistance of OCSCs to ADR by affecting the expression and transport function of BCRP.

### 3.5 HIF‐2α directly activates expression of the BCRP gene in ovarian cancer cells

**Figure 5:**
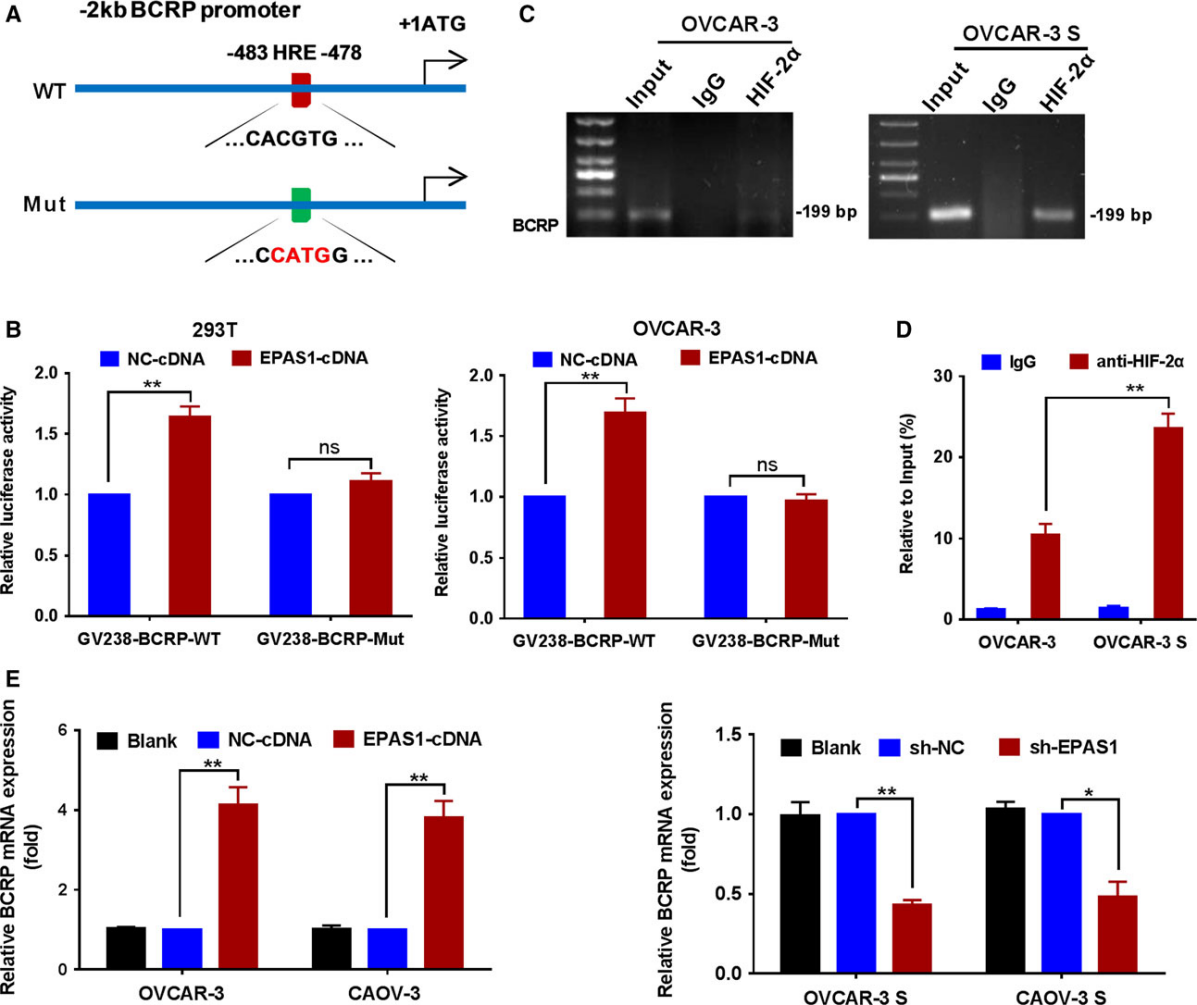
HIF‐2α directly activates the *BCRP* gene in ovarian cancer cells. (A) The evolutionarily conserved hypoxia‐response element (HRE) sequence (CACGTG), located between −483 and −478 nucleotides upstream of the transcription start site of the human *BCRP* gene. (B) Luciferase reporter activity was measured in transient co‐transfections with *EPAS1*‐cDNA or NC‐cDNA plasmids and *BCRP* promoter vectors (GV238‐BCRP‐WT) or vectors containing mutated HRE sequence binding sites (GV238‐BCRP‐mut) in 293T and OVCAR‐3 cells. The luciferase activities in the cells co‐transfected with NC‐cDNA and GV238‐BCRP‐WT or GV238‐BCRP‐mut plasmids were set as 1. Data are presented as the mean ± SD from three independent experiments. (C) ChIP assays were performed to verify HIF‐2α binding to the *BCRP* gene in OVCAR‐3 and OVCAR‐3 S cells cultured under hypoxic conditions (1% O_2_) for 48 h. (D) ChIP‐qPCR shows the enhanced binding HIF‐2α on BCRP promoter in OVCAR‐3 S cells. Antibody enrichment was quantified relative to the amount of input DNA. Antibody directed against IgG was used as a negative control. (E) The mRNA expression of BCRP was analyzed by qRT‐PCR in the OVCAR‐3 and CAOV‐3 cells transduced with *EPAS1*‐cDNA or NC‐cDNA lentivirus, and in the OVCAR‐3 S and CAOV‐3 S cells transduced with sh‐*EPAS1* or sh‐NC lentivirus under hypoxic conditions (1% O_2_) for 48 h. BCRP expression levels in OVCAR‐3 cells or CAOV‐3 cells transduced with *EPAS1*‐cDNA lentivirus and in OVCAR‐3 S cells or CAOV‐3 S cells transduced with sh‐NC lentivirus were set as 1. Data are presented as the mean ± SD from three independent experiments. *P <* *0.05, **P <* *0.01. Statistical significance was evaluated by Student's *t*‐test.

To further study the exact mechanism by which HIF‐2α regulates the expression and function of BCRP, we first analyzed sequences in 2‐kb upstream of the *BCRP* gene. We found that there was an evolutionarily conserved hypoxia‐response element (HRE) sequence (CACGTG) located between −483 and −478 bases upstream of the transcription start site of the *BCRP* gene (positions 88158941–88158936 in *BCRP* gene sequence (GenBank accession number NM_001257386.2, Fig. 5A)). Next, we utilized BCRP promoter vectors (GV238‐BCRP‐WT) or vectors with mutated HRE sequence binding sites (GV238‐BCRP‐mut) to drive a luciferase reporter gene in transient co‐transfections with HIF‐2α‐cDNA plasmids in 293T and OVCAR‐3 cells. We found that ectopic expression of HIF‐2α strongly increased luciferase activity in cells transfected with GV238‐BCRP‐WT plasmids, but not in cells transfected with GV238‐BCRP‐mut plasmids (Fig. 5B).

We also determined whether HIF‐2α bound to the BCRP promoter *in vivo* using ChIP assays. As shown in Fig. 5C, chromatin solutions prepared from OVCAR‐3 and OVCAR‐3 S cells were used to immunoprecipitate the HIF‐2α‐DNA complex using anti‐HIF‐2α serum or control IgG serum followed by PCR amplification of the *BCRP* gene sequence. Using primers designed to amplify the HRE, we were able to amplify product from the DNA bound to protein precipitated with the anti‐HIF‐2α serum, but not from DNA precipitated using control antibodies, indicating the specificity of HIF‐2α binding to the BCRP promoter. Moreover, we found that HIF‐2α highly bound BCRP gene promoter region in OVCAR‐3 S cells compared to that in parental cells (Fig. 5C) because OVCAR‐3 S cells have higher expression of HIF‐2α than OVCAR‐3 cells (Fig. S4A). ChIP‐qPCR further demonstrated the enhanced binding HIF‐2α on BCRP promoter in OVCAR‐3 S cells (Fig. 5D). Additionally, we also performed ChIP assay of HIF‐1α binding with VEGF‐HRE and BCRP‐HRE in OVCAR‐3 and OVCAR‐3 S cells, and found the binding of HIF‐1α with VEGF‐HRE, but no binding with BCRP‐HRE (Fig. S9A,B). Thus, the results suggest that BCRP promoter is targeted by HIF‐2α but not by HIF‐1α in OVCAR‐3 cells.

Next, we determined the BCRP mRNA expression in the HIF‐2α‐overexpressing OVCAR‐3 and CAOV‐3 cells and in the OVCAR‐3 S and CAOV‐3 S cells transduced with sh‐*EPAS1* lentivirus using qRT‐PCR. HIF‐2α overexpression significantly upregulated the BCRP mRNA expression in the OVCAR‐3 and CAOV‐3 cells, while HIF‐2α depletion significantly reduced BCRP mRNA expression levels in OVCAR‐3 S and CAOV‐3 S cells (Fig. 5E). Collectively, the data confirm that HIF‐2α can directly activate *BCRP* gene expression in ovarian cancer cells.

### 3.6 Silencing HIF‐2α inhibits the growth of OCSCs *in vivo*

**Figure 6:**
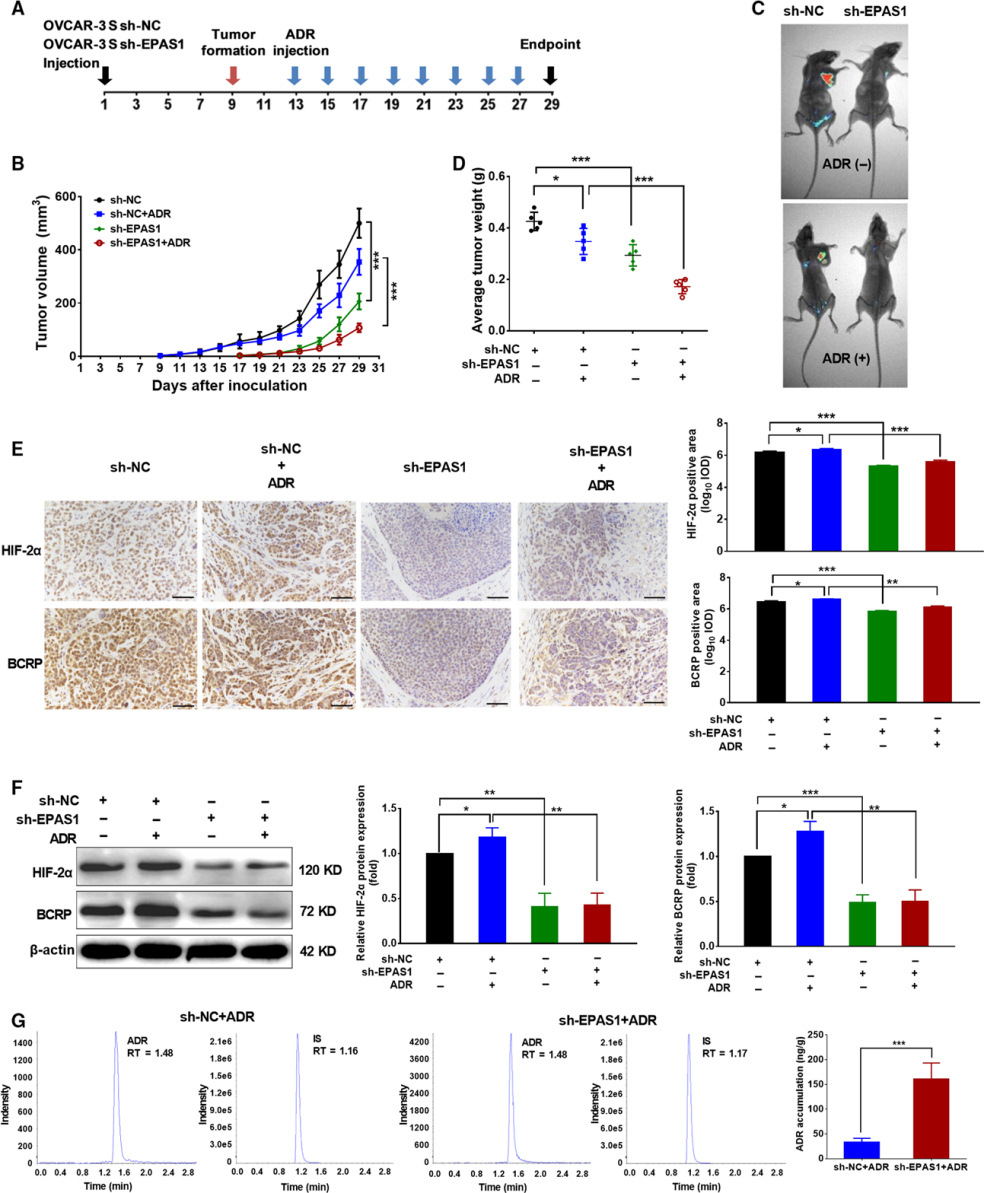
Silencing HIF‐2α increases the response of OCSCs to ADR *in vivo*. (A) A diagram showing the time of tumor formation in BALB/c mice transplanted with the OVCAR‐3 S cells (1 × 10^6^) stably transfected sh‐*EPAS1* lentiviral transduction particles or sh‐NC as a control and after ADR or control DMSO intraperitoneal injection *in vivo* (*n *=* *5 mice in each group). (B) Growth curves of tumor volumes measured every other day in xenograft mice. Error bars indicate SD. ***P <* *0.001. (C) GFP expression was detected in xenograft mice by small animal imaging. (D) The tumor weight was compared after sacrifice of xenograft mice 4 weeks after cell inoculation. Error bars indicate SD. *P <* *0.05, ***P <* *0.001. (E) Representative immunohistochemical staining for the expression of HIF‐2α and BCRP proteins and statistical results for IOD in the xenograft tumors. Error bars indicate SD. *P <* *0.05, **P <* *0.01, ***P <* *0.001. The length of the scale bars is 20 μm. (F) Representative images for the expression of HIF‐2α and BCRP proteins in the xenograft tumors by western blot and the relative protein expression of HIF‐2α and BCRP normalized to those of β‐actin were analyzed. The expression levels of HIF‐2α/BCRP in the xenograft tumors derived from cells transduced with sh‐NC and without ADR treatment were set as 1. Error bars indicate SD. *P <* *0.05, **P <* *0.01, ***P <* *0.001. (G) ADR accumulation in xenograft tumors derived from sh‐*EPAS1* or sh‐NC transduced OVCAR‐3 S cells by mass spectrometry. The representative chromatograms were for ADR, internal standard (IS) in the sh‐NC xenograft tumors with ADR treatment, and ADR, IS in the sh‐*EPAS1* xenograft tumors with ADR treatment. RT, Retention Time. Error bars indicate SD. ***P <* *0.001. Statistical significance was evaluated by one‐way anova.

To further investigate whether HIF‐2α could mediate the response of OCSCs to ADR by regulating BCRP *in vivo*, we used the BALB/c (nu/nu) mouse model to evaluate the effects of HIF‐2α expression changes on the sensitivity of OCSCs to ADR. OVCAR‐3 S cells (1 × 10^6^) stably transfected with sh‐*EPAS1* lentiviral transduction particles, or sh‐NC as a control, were subcutaneously injected into nude mice. Thirteen days after inoculation, transplanted nude mice were intraperitoneally injected with ADR (1.5 mg·kg^−1^) or DMSO as a control once every other day, respectively (Fig. 6A). We observed that the tumor volumes and tumor weights in the xenografted mice transplanted with sh‐*EPAS1*‐transduced OVCAR‐3 S cells were both significantly reduced compared with those in sh‐NC xenografted mice. Additionally, ADR treatment showed weakly inhibitory effects on the tumor volumes and tumor weights in sh‐NC xenografted mice (*P *<* *0.05), and tumor growth was notably decreased in sh‐*EPAS1* xenografted mice treated with ADR (*P *<* *0.001, Fig. 6B–D). These findings demonstrate that decreased HIF‐2α could inhibit the growth of OCSC *in vivo*.

To further evaluate the association between the expression of HIF‐2α and BCRP *in vivo*, we measured HIF‐2α and BCRP mRNA expression using RT‐PCR and protein expression using immunohistochemical staining and western blot of the transplanted tumors. As shown in Figs 6E,F and S10A,B, the mRNA and protein expression levels of HIF‐2α and BCRP were significantly reduced in the transplanted tumors transduced with sh‐*EPAS1* lentivirus as compared with those transduced with sh‐NC lentivirus. In addition, mRNA and protein expression levels of HIF‐2α and BCRP were significantly decreased in the sh‐*EPAS1*‐transduced mice treated with ADR as compared to those in the sh‐NC‐transduced mice treated with ADR. We also evaluated the effects of silencing HIF‐2α on the ability of BCRP to transport ADR *in vivo* using mass spectrometry. As expected, our results indicated that the ADR accumulation in sh‐*EPAS1*‐transduced OVCAR‐3 S xenograft tissue was much higher than the sh‐NC‐transduced OVCAR‐3 S xenograft tissue (Fig. 6G). Thus, these data suggest that silencing HIF‐2α can inhibit the growth of OCSC by decreasing the expression and function of BCRP *in vivo*.

## 4 Discussion

OCSCs are believed to be the cause of ovarian cancer chemoresistance and recurrence (Jung *et al*., 2016). However, the mechanisms underlying OCSC‐mediated chemoresistance remain unclear. OCSCs were first reported as multilayered spheroids, displaying heterogeneity and stem‐like qualities, and were derived from the ascites of a patient with advanced ovarian cancer (Bapat *et al*., 2005). In the present study, we obtained OVCAR‐3 and CAOV‐3 sphere‐forming cells (OVCAR‐3 S and CAOV‐3 S) by culturing OVCAR‐3 and CAOV‐3 cells in suspension in serum‐free medium as previously reported by Wang *et al*. (2012). OVCAR‐3 S and CAOV‐3 S cells exhibited OCSC‐like characteristics such as high expression of the stem cell markers OCT4 and nanog, strong colony formation ability, increased invasion capability, and strong tumorigenicity *in vivo*. CD133, a cellular surface protein, has been reported as a potential OCSC marker (Curley *et al*., 2009). In addition, the high enzymatic activity of aldehyde dehydrogenase (ALDH) was also defined as a marker for various CSCs, including OCSCs (Silva *et al*., 2011). In our study, OVCAR‐3 S and CAOV‐3 S cells exhibited increased expression of CD133 and higher enzymatic activity of ALDH relative to the parental OVCAR‐3 and CAOV‐3 cells, which was consistent with Wang *et al*.'s (2012) previous reports. Importantly, we found that OVCAR‐3 S and CAOV‐3 S cells exhibited reduced sensitivity to the anticancer drugs ADR, MX, PTX, VP‐16, and DDP as compared with their parental cells. The RI values of OVCAR‐3 S cells relative to OVCAR‐3 cells were 103.6, 20.9, 17.3, 24.9, and 3.55, respectively, and the RI values of CAOV‐3 S cell relative to CAOV‐3 cells were 24.7, 14.1, 12.8, 15.9, and 3.33, respectively. Overall, OVCAR‐3 S and CAOV‐3 S cells showed the strongest resistance to ADR. Meirelles *et al*. reported that the OCSCs isolated from OVCAR‐7 ovarian cancer cell lines were resistant to ADR (Meirelles *et al*., 2012). Therefore, the OVCAR‐3 S and CAOV‐3 S cells in the present study can be reliably used to investigate the effects and mechanisms of the resistance to ADR on OCSCs.

Hypoxic microenvironments have been identified in many solid tumors and increase CSC characteristics of tumor cells, including the resistance to chemotherapeutic agents (Axelson *et al*., 2005). In this study, we used the TCGA data and an online tool, KM plotter analysis, to identify the positive relationship between HIF‐2α (not HIF‐1α) and ALDH1A1 mRNA expression as well as the positive association between higher HIF‐2α (but not HIF‐1α) mRNA expression and shorter OS in ovarian cancer patients. In addition, we found a significant positive correlation between HIF‐2α and ALDH1A1/CD133 protein expression, but only a weak positive correlation of HIF‐1α and ALDH1A1 protein expression in 115 ovarian tumor tissue samples. These findings suggest that HIF‐2α may be related to stem‐like characteristics of OCSC and poor outcome in ovarian cancer patients. It has been reported that high expression of HIF‐2α protein is correlated with ALDH activity in breast cancer cell lines and tissues (Kim *et al*., 2013). Other groups reported that HIF‐1α expression was positively correlated with the expression of CD133 in renal cell carcinoma (RCC) tissues (Sun *et al*., 2012) and with the ALDH1 expression in astrocytoma (Inukai *et al*., 2015). By contrast, our findings suggest that HIF‐2α, rather than HIF‐1α, has a greater correlation with OCSC maintenance.

In this study, we report that hypoxia increased the resistance of OVCAR‐3 S and CAOV‐3 S cells to ADR. We aimed to determine whether HIF‐1α or HIF‐2α play major roles in the resistance of OCSC to ADR. We found that hypoxia (1% O_2_) especially increased the expression of HIF‐2α in the OVCAR‐3 S and CAOV‐3 S cells. Importantly, treatment with ADR significantly induced higher expression of HIF‐2α, but not HIF‐1α, protein in both OVCAR‐3 S and CAOV‐3 S cells, suggesting a role of HIF‐2α in OCSC resistance to ADR. Furthermore, our study found that *HIF‐2*α silencing in OVCAR‐3 S and CAOV‐3 S cells dramatically reduced ADR resistance and colony formation ability, while overexpression of HIF‐2α in OVCAR‐3 and CAOV‐3 cells resulted in resistance to ADR and reduced colony formation ability, indicating HIF‐2α may be a master regulator of ADR resistance in OCSCs. Seo *et al*. reported that the hypoxia‐NOTCH1‐SOX2 signaling axis was important for maintaining ‘stemness’‐related characteristics in OCSCs (Seo *et al*., 2016). Although they found that deletion of HIF‐1 inhibited the increased SOX2 promoter activity induced by hypoxia, the expression and function of HIF‐2α were not measured in their study. Our study mainly focused on the effects and mechanisms of HIF‐2α in regulating ADR resistance of OCSCs, which has not yet been reported in the literature thus far.

Overexpression of BCRP, a key member of the ATP‐binding cassette transporter family and a major CSC marker, contributes to chemotherapeutic drug resistance in CSCs (Bunting, 2002), including OCSCs (Januchowski *et al*., 2016). Xiang *et al*. reported that the expression of HIF‐2α protein was positively correlated with expression of the BCRP protein in breast cancer tissues (Xiang *et al*., 2012). In our study, we found that both mRNA and protein expression of HIF‐2α were positively associated with mRNA and protein expression of BCRP by TCGA data analysis and in ovarian cancer tissue samples. Furthermore, HIF‐2α depletion significantly reduced the mRNA and protein expression of BCRP and enhanced the cellular accumulation of ADR in OVCAR‐3 S and CAOV‐3 S cells. Similarly, HIF‐2α overexpression significantly increased the mRNA and protein expression of BCRP and decreased the cellular accumulation of ADR in the OVCAR‐3 and CAOV‐3 cells. Moreover, HIF‐2α silencing significantly reduced mRNA and protein expression of BCRP and enhanced cellular accumulation of ADR in OVCAR‐3 S xenograft tumors. These results suggest a possible relationship between HIF‐2α and BCRP in the regulation of OCSC resistance to ADR. Chen *et al*. reported that knockdown BCRP could inhibit cell proliferation of MCF‐7/MX by inhibiting S phase of cell cycle and the expression of cyclin D3 (Chen *et al*., 2010). We speculate that the inhibitory effects of silencing *EPAS1* without ADR treatment on tumor growth may be related to the function of BCRP. Besides, HIF‐2α may play the effects of inhibiting cell proliferation and stemness phenotype by targeting other genes, such as OCT4 we have measured.

It has been reported that HIF‐2α binds to the HREs of target genes and activates these genes, including Epo (Warnecke *et al*., 2004) and fetal liver kinase‐1 (Flk‐1) (Takeda *et al*., 2004). We found that there was an evolutionarily conserved HRE sequence (CACGTG) from ‐483 to ‐478 bases in the 2‐kb upstream region of the *BCRP* gene. Also, HIF‐2α could directly activate BCRP via the HRE binding site in ovarian cancer cells. We also demonstrated the HIF‐2α could bind to the *BCRP* promoter *in vivo* via ChIP assay. Martin *et al*. reported that HIF‐2α bound to an evolutionary conserved HRE in the murine *BCRP* promoter (Martin *et al*., 2008). In contrast to these reports, we found a novel HRE binding site for HIF‐2α in the human *BCRP* promoter and demonstrated its transactivation in human ovarian cancer cells.

## 5 Conclusion

In general, our studies supply a novel mechanism whereby HIF‐2α regulates the resistance of OCSCs to ADR by directly activating BCRP. Moreover, our findings suggest that HIF‐2α may represent an important target for reversing OCSC‐mediated chemoresistance and HIF‐2α inhibitors in combination with conventional chemotherapy are expected to improve therapeutic outcomes in ovarian cancer patients.

## Conflict of interest

The authors declare no conflict of interest.

## Author contributions

MW and MH designed the experiments. HW, QJ, YL, LH, BW, YY, FL, XD, HC, WY, HZ, and MH performed the experiments. QJ, HW, and MH analyzed the data. MW and XW gave material support. MH, MW, LZ, and JC wrote and reviewed the manuscript.

## Supporting information


**Fig. S1.** OVCAR‐3 S and CAOV‐3 S cells possess OCSC‐like properties.

**Fig. S2.** Ovarian cancer sphere‐forming cells, OVCAR‐3 S and CAOV‐3 S, are resistant to chemotherapeutic drugs.

**Fig. S3.** Representative pictures of the protein expression of HIF‐1α‐negative (HIF‐1α(‐)), HIF‐1α‐positive (HIF‐1α(+)), HIF‐2α‐negative (HIF‐2α(‐)), HIF‐2α‐positive (HIF‐2α(+)), CD133‐negative (CD133(‐)), CD133‐positive (CD133(+)), ALDHA1‐negative (ALDHA1(‐)), and ALDHA1‐positive (ALDHA1(+)) staining in 115 ovarian tumor tissues using immunohistochemistry. (Related to Fig. 2).

**Fig. S4.** The expression changes of HIF‐1α or HIF‐2α in OVCAR‐3 S and CAOV‐3 S cells and hypoxia‐treated OVCAR‐3 and CAOV‐3 cells.

**Fig. S5.** The transduction efficiency of OVCAR‐3 S and CAOV‐3 S cells with silenced *HIF‐1*α or *HIF‐2*α.

**Fig. S6.** The transduction efficiency of OVCAR‐3 and CAOV‐3 cells overexpressing HIF‐1α or HIF‐2α.

**Fig. S7.** The effects of overexpression of HIF‐1α or HIF‐2α on the protein expression of OCT4 in OVCAR‐3 and CAOV‐3 cells.

**Fig. S8.** The effects of the HIF‐2α on the intracellular accumulation of ADR in ovarian cancer cells by mass spectrometry.

**Fig. S9.** The determination of HIF‐1α binding to the VEGF and BCRP promoter.

**Fig. S10.** The effects of silencing *HIF‐2*α on the expression of BCRP in OCSCs xenograft mice.

**Table S1.** Clinicopathological features of 115 ovarian cancer patients.

## References

[mol212419-bib-0001] Ai ZH , Lu Y , Qiu SB and Fan Z (2016) Overcoming cisplatin resistance of ovarian cancer cells by targeting HIF‐1‐regulated cancer metabolism. Cancer Lett 373, 36–44. 26801746 10.1016/j.canlet.2016.01.009 PMC4769873

[mol212419-bib-0002] Axelson H , Fredlund E , Ovenberger M , Landberg G and Pahlman S (2005) Hypoxia‐induced dedifferentiation of tumor cells–a mechanism behind heterogeneity and aggressiveness of solid tumors. Semin Cell Dev Biol 16, 554–563. 16144692 10.1016/j.semcdb.2005.03.007

[mol212419-bib-0003] Bapat SA , Mali AM , Koppikar CB and Kurrey NK (2005) Stem and progenitor‐like cells contribute to the aggressive behavior of human epithelial ovarian cancer. Cancer Res 65, 3025–3029. 15833827 10.1158/0008-5472.CAN-04-3931

[mol212419-bib-0004] Baran N and Konopleva M (2017) Molecular pathways: hypoxia‐activated prodrugs in cancer therapy. Clin Cancer Res 23, 2382–2390. 28137923 10.1158/1078-0432.CCR-16-0895 PMC5433896

[mol212419-bib-0005] Bunting KD (2002) ABC transporters as phenotypic markers and functional regulators of stem cells. Stem Cells 20, 11–20. 11796918 10.1002/stem.200011

[mol212419-bib-0006] Chen Z , Liu F , Ren Q , Zhao Q , Ren H , Lu S , Zhang L and Han Z (2010) Suppression of ABCG2 inhibits cancer cell proliferation. Int J Cancer 126, 841–851. 19642144 10.1002/ijc.24796

[mol212419-bib-0007] Chen W , Zheng R , Baade PD , Zhang S , Zeng H , Bray F , Jemal A , Yu XQ and He J (2016) Cancer statistics in China, 2015. CA Cancer J Clin 66, 115–132. 26808342 10.3322/caac.21338

[mol212419-bib-0008] Curley MD , Therrien VA , Cummings CL , Sergent PA , Koulouris CR , Friel AM , Roberts DJ , Seiden MV , Scadden DT , Rueda BR *et al* (2009) CD133 expression defines a tumor initiating cell population in primary human ovarian cancer. Stem Cells 27, 2875–2883. 19816957 10.1002/stem.236

[mol212419-bib-0009] Doyle LA , Yang W , Abruzzo LV , Krogmann T , Gao Y , Rishi AK and Ross DD (1998) A multidrug resistance transporter from human MCF‐7 breast cancer cells. Proc Natl Acad Sci U S A 95, 15665–15670. 9861027 10.1073/pnas.95.26.15665 PMC28101

[mol212419-bib-0010] Fu YZ , Yan YY , He M , Xiao QH , Yao WF , Zhao L , Wu HZ , Yu ZJ , Zhou MY , Lv MT *et al* (2016) Salinomycin induces selective cytotoxicity to MCF‐7 mammosphere cells through targeting the Hedgehog signaling pathway. Oncol Rep 35, 912–922. 26718029 10.3892/or.2015.4434

[mol212419-bib-0011] Gilani RA , Kazi AA , Shah P , Schech AJ , Chumsri S , Sabnis G , Jaiswal AK and Brodie AH (2012) The importance of HER2 signaling in the tumor‐initiating cell population in aromatase inhibitor‐resistant breast cancer. Breast Cancer Res Treat 135, 681–692. 22878889 10.1007/s10549-012-2148-8

[mol212419-bib-0012] He M , Fu Y , Yan Y , Xiao Q , Wu H , Yao W , Zhao H , Zhao L , Jiang Q , Yu Z *et al* (2015) The Hedgehog signalling pathway mediates drug response of MCF‐7 mammosphere cells in breast cancer patients. Clin Sci (Lond) 129, 809–822. 26201092 10.1042/CS20140592

[mol212419-bib-0013] Heddleston JM , Li Z , Lathia JD , Bao S , Hjelmeland AB and Rich JN (2010) Hypoxia inducible factors in cancer stem cells. Br J Cancer 102, 789–795. 20104230 10.1038/sj.bjc.6605551 PMC2833246

[mol212419-bib-0014] Hu L , McArthur C and Jaffe RB (2010) Ovarian cancer stem‐like side‐population cells are tumourigenic and chemoresistant. Br J Cancer 102, 1276–1283. 20354527 10.1038/sj.bjc.6605626 PMC2856005

[mol212419-bib-0015] Inukai M , Hara A , Yasui Y , Kumabe T , Matsumoto T and Saegusa M (2015) Hypoxia‐mediated cancer stem cells in pseudopalisades with activation of hypoxia‐inducible factor‐1alpha/Akt axis in glioblastoma. Hum Pathol 46, 1496–1505. 26256949 10.1016/j.humpath.2015.06.008

[mol212419-bib-0016] Januchowski R , Wojtowicz K , Sterzynska K , Sosinska P , Andrzejewska M , Zawierucha P , Nowicki M and Zabel M (2016) Inhibition of ALDH1A1 activity decreases expression of drug transporters and reduces chemotherapy resistance in ovarian cancer cell lines. Int J Biochem Cell Biol 78, 248–259. 27443528 10.1016/j.biocel.2016.07.017

[mol212419-bib-0017] Jung KA , Choi BH and Kwak MK (2015) The c‐MET/PI3K signaling is associated with cancer resistance to doxorubicin and photodynamic therapy by elevating BCRP/ABCG2 expression. Mol Pharmacol 87, 465–476. 25534417 10.1124/mol.114.096065

[mol212419-bib-0018] Jung JG , Shih IM , Park JT , Gerry E , Kim TH , Ayhan A , Handschuh K , Davidson B , Fader AN , Selleri L *et al* (2016) Ovarian cancer chemoresistance relies on the stem cell reprogramming factor PBX1. Cancer Res 76, 6351–6361. 27590741 10.1158/0008-5472.CAN-16-0980 PMC7375390

[mol212419-bib-0019] Kim RJ , Park JR , Roh KJ , Choi AR , Kim SR , Kim PH , Yu JH , Lee JW , Ahn SH , Gong G *et al* (2013) High aldehyde dehydrogenase activity enhances stem cell features in breast cancer cells by activating hypoxia‐inducible factor‐2alpha. Cancer Lett 333, 18–31. 23174107 10.1016/j.canlet.2012.11.026

[mol212419-bib-0020] Koh MY and Powis G (2012) Passing the baton: the HIF switch. Trends Biochem Sci 37, 364–372. 22818162 10.1016/j.tibs.2012.06.004 PMC3433036

[mol212419-bib-0021] Ma MT , He M , Wang Y , Jiao XY , Zhao L , Bai XF , Yu ZJ , Wu HZ , Sun ML , Song ZG *et al* (2013) MiR‐487a resensitizes mitoxantrone (MX)‐resistant breast cancer cells (MCF‐7/MX) to MX by targeting breast cancer resistance protein (BCRP/ABCG2). Cancer Lett 339, 107–115. 23879965 10.1016/j.canlet.2013.07.016

[mol212419-bib-0022] Ma W , Wang J , Guo Q and Tu P (2015) Quantitative subcellular study of doxorubicin in MCF‐7/Adr cells using liquid chromatography‐tandem mass spectrometry. J Chromatogr B Analyt Technol Biomed Life Sci 1007, 18–22. 10.1016/j.jchromb.2015.11.002 26562803

[mol212419-bib-0023] Martin CM , Ferdous A , Gallardo T , Humphries C , Sadek H , Caprioli A , Garcia JA , Szweda LI , Garry MG and Garry DJ (2008) Hypoxia‐inducible factor‐2alpha transactivates Abcg2 and promotes cytoprotection in cardiac side population cells. Circ Res 102, 1075–1081. 18356544 10.1161/CIRCRESAHA.107.161729

[mol212419-bib-0024] Maugeri‐Sacca M , Vigneri P and De Maria R (2011) Cancer stem cells and chemosensitivity. Clin Cancer Res 17, 4942–4947. 21622723 10.1158/1078-0432.CCR-10-2538

[mol212419-bib-0025] Meirelles K , Benedict LA , Dombkowski D , Pepin D , Preffer FI , Teixeira J , Tanwar PS , Young RH , MacLaughlin DT , Donahoe PK *et al* (2012) Human ovarian cancer stem/progenitor cells are stimulated by doxorubicin but inhibited by Mullerian inhibiting substance. Proc Natl Acad Sci U S A 109, 2358–2363. 22308459 10.1073/pnas.1120733109 PMC3289387

[mol212419-bib-0026] Mullendore ME , Koorstra JB , Li YM , Offerhaus GJ , Fan X , Henderson CM , Matsui W , Eberhart CG , Maitra A and Feldmann G (2009) Ligand‐dependent Notch signaling is involved in tumor initiation and tumor maintenance in pancreatic cancer. Clin Cancer Res 15, 2291–2301. 19258443 10.1158/1078-0432.CCR-08-2004 PMC2711441

[mol212419-bib-0027] Natarajan K , Xie Y , Baer MR and Ross DD (2012) Role of breast cancer resistance protein (BCRP/ABCG2) in cancer drug resistance. Biochem Pharmacol 83, 1084–1103. 22248732 10.1016/j.bcp.2012.01.002 PMC3307098

[mol212419-bib-0028] Ottevanger PB (2017) Ovarian cancer stem cells more questions than answers. Semin Cancer Biol 44, 67–71. 28450177 10.1016/j.semcancer.2017.04.009

[mol212419-bib-0029] Pietras A , Hansford LM , Johnsson AS , Bridges E , Sjolund J , Gisselsson D , Rehn M , Beckman S , Noguera R , Navarro S *et al* (2009) HIF‐2alpha maintains an undifferentiated state in neural crest‐like human neuroblastoma tumor‐initiating cells. Proc Natl Acad Sci U S A 106, 16805–16810. 19805377 10.1073/pnas.0904606106 PMC2745331

[mol212419-bib-0030] Ponti D , Costa A , Zaffaroni N , Pratesi G , Petrangolini G , Coradini D , Pilotti S , Pierotti MA and Daidone MG (2005) Isolation and *in vitro* propagation of tumorigenic breast cancer cells with stem/progenitor cell properties. Cancer Res 65, 5506–5511. 15994920 10.1158/0008-5472.CAN-05-0626

[mol212419-bib-0031] Seo EJ , Kim DK , Jang IH , Choi EJ , Shin SH , Lee SI , Kwon SM , Kim KH , Suh DS and Kim JH (2016) Hypoxia‐NOTCH1‐SOX2 signaling is important for maintaining cancer stem cells in ovarian cancer. Oncotarget 7, 55624–55638. 27489349 10.18632/oncotarget.10954 PMC5342441

[mol212419-bib-0032] Siegel RL , Miller KD and Jemal A (2017) Cancer statistics, 2017. CA Cancer J Clin 67, 7–30. 28055103 10.3322/caac.21387

[mol212419-bib-0033] Silva IA , Bai S , McLean K , Yang K , Griffith K , Thomas D , Ginestier C , Johnston C , Kueck A , Reynolds RK *et al* (2011) Aldehyde dehydrogenase in combination with CD133 defines angiogenic ovarian cancer stem cells that portend poor patient survival. Cancer Res 71, 3991–4001. 21498635 10.1158/0008-5472.CAN-10-3175 PMC3107359

[mol212419-bib-0034] Sun C , Song H , Zhang H , Hou C , Zhai T , Huang L and Zhang L (2012) CD133 expression in renal cell carcinoma (RCC) is correlated with nuclear hypoxia‐inducing factor 1alpha (HIF‐1alpha). J Cancer Res Clin Oncol 138, 1619–1624. 22614155 10.1007/s00432-012-1237-8 PMC11824241

[mol212419-bib-0035] Szotek PP , Pieretti‐Vanmarcke R , Masiakos PT , Dinulescu DM , Connolly D , Foster R , Dombkowski D , Preffer F , Maclaughlin DT and Donahoe PK (2006) Ovarian cancer side population defines cells with stem cell‐like characteristics and Mullerian Inhibiting Substance responsiveness. Proc Natl Acad Sci U S A 103, 11154–11159. 16849428 10.1073/pnas.0603672103 PMC1544057

[mol212419-bib-0036] Takakura N (2012) Formation and regulation of the cancer stem cell niche. Cancer Sci 103, 1177–1181. 22416970 10.1111/j.1349-7006.2012.02270.x PMC7659329

[mol212419-bib-0037] Takeda N , Maemura K , Imai Y , Harada T , Kawanami D , Nojiri T , Manabe I and Nagai R (2004) Endothelial PAS domain protein 1 gene promotes angiogenesis through the transactivation of both vascular endothelial growth factor and its receptor, Flt‐1. Circ Res 95, 146–153. 15192019 10.1161/01.RES.0000134920.10128.b4

[mol212419-bib-0038] Vermeersch KA , Wang L , Mezencev R , McDonald JF and Styczynski MP (2015) OVCAR‐3 spheroid‐derived cells display distinct metabolic profiles. PLoS ONE 10, e0118262. 25688563 10.1371/journal.pone.0118262 PMC4331360

[mol212419-bib-0039] Wang L , Mezencev R , Bowen NJ , Matyunina LV and McDonald JF (2012) Isolation and characterization of stem‐like cells from a human ovarian cancer cell line. Mol Cell Biochem 363, 257–268. 22160925 10.1007/s11010-011-1178-6

[mol212419-bib-0040] Warnecke C , Zaborowska Z , Kurreck J , Erdmann VA , Frei U , Wiesener M and Eckardt KU (2004) Differentiating the functional role of hypoxia‐inducible factor (HIF)‐1alpha and HIF‐2alpha (EPAS‐1) by the use of RNA interference: erythropoietin is a HIF‐2alpha target gene in Hep3B and Kelly cells. FASEB J 18, 1462–1464. 15240563 10.1096/fj.04-1640fje

[mol212419-bib-0041] Wu MJ , Jan CI , Tsay YG , Yu YH , Huang CY , Lin SC , Liu CJ , Chen YS , Lo JF and Yu CC (2010) Elimination of head and neck cancer initiating cells through targeting glucose regulated protein78 signaling. Mol Cancer 9, 283. 20979610 10.1186/1476-4598-9-283 PMC2987982

[mol212419-bib-0042] Xiang L , Liu ZH , Huan Q , Su P , Du GJ , Wang Y , Gao P and Zhou GY (2012) Hypoxia‐inducible factor‐2a is associated with ABCG2 expression, histology‐grade and Ki67 expression in breast invasive ductal carcinoma. Diagn Pathol 7, 32. 22452996 10.1186/1746-1596-7-32 PMC3337233

[mol212419-bib-0043] Zhao L , Wang Y , Jiang L , He M , Bai X , Yu L and Wei M (2016) MiR‐302a/b/c/d cooperatively sensitizes breast cancer cells to adriamycin via suppressing P‐glycoprotein (P‐gp) by targeting MAP/ERK kinase kinase 1 (MEKK1). J Exp Clin Cancer Res 35, 25. 26842910 10.1186/s13046-016-0300-8 PMC4738800

[mol212419-bib-0044] Zhao D , Zhai B , He C , Tan G , Jiang X , Pan S , Dong X , Wei Z , Ma L , Qiao H *et al* (2014) Upregulation of HIF‐2alpha induced by sorafenib contributes to the resistance by activating the TGF‐alpha/EGFR pathway in hepatocellular carcinoma cells. Cell Signal 26, 1030–1039. 24486412 10.1016/j.cellsig.2014.01.026

