# Supplementary figures and images for: Hypoxia‐inducible factor‐2α directly promotes BCRP expression and mediates the resistance of ovarian cancer stem cells to adriamycin
Source: Mol Oncol. 2019 Jan 14;13(2):403–21. doi: 10.1002/1878-0261.12419 (PMC6360369; doi:10.1002/1878-0261.12419)

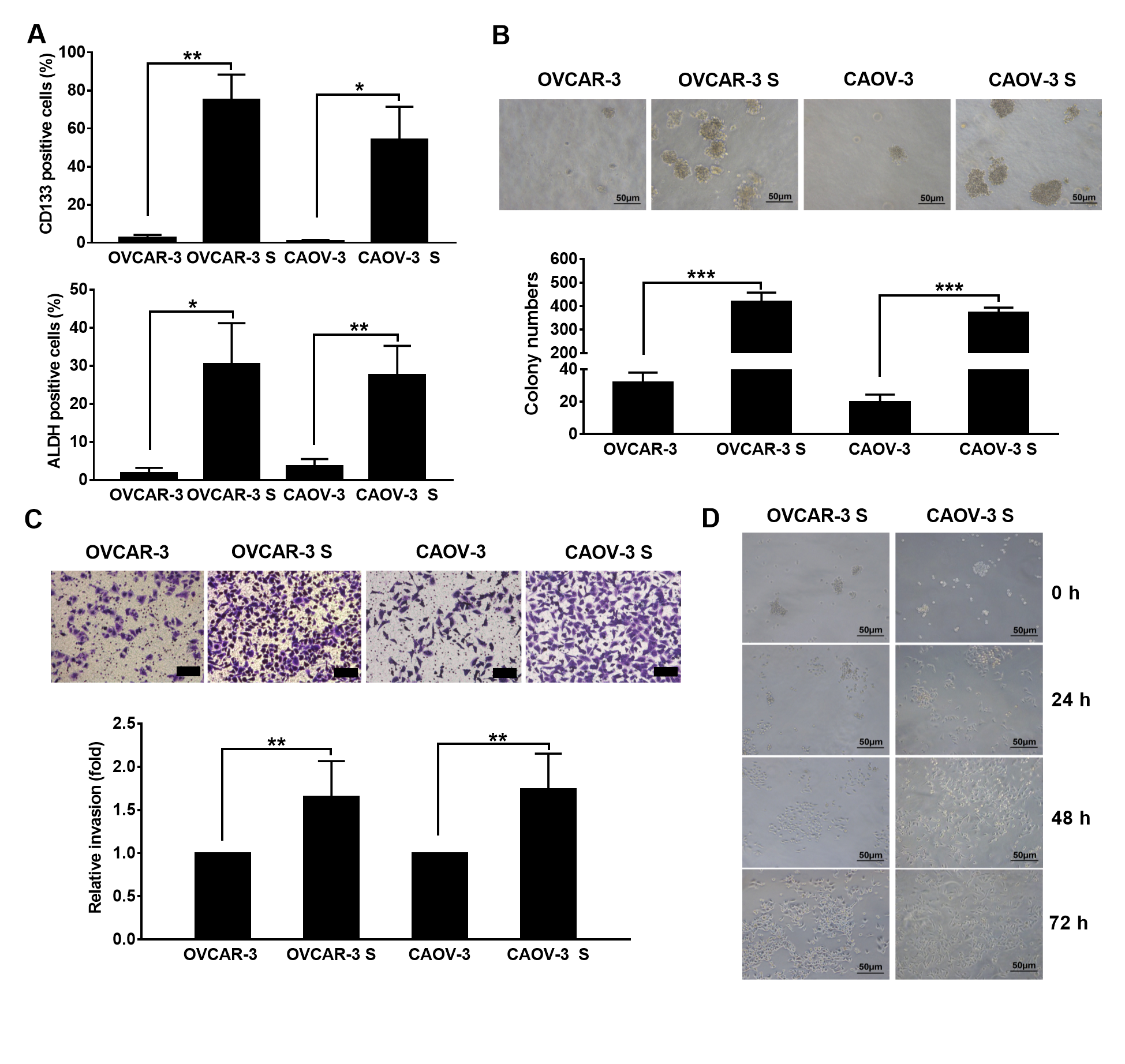

Supplement: Supplementary file 1 — Fig. S1. OVCAR‐3 S and CAOV‐3 S cells possess OCSC‐like properties. [file MOL2-13-403-s001.tif]

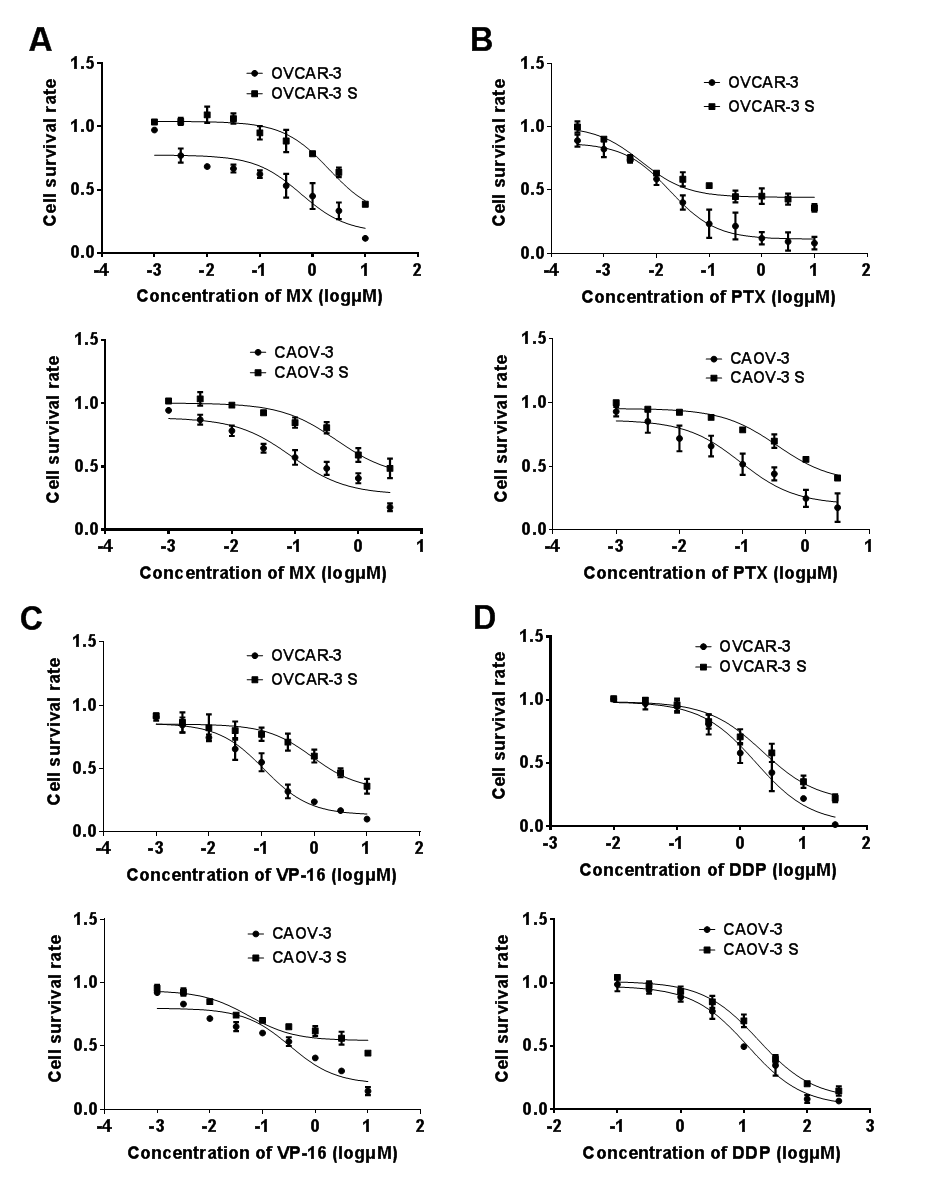

Supplement: Supplementary file 2 — Fig. S2. Ovarian cancer sphere‐forming cells, OVCAR‐3 S and CAOV‐3 S, are resistant to chemotherapeutic drugs. [file MOL2-13-403-s002.tif]

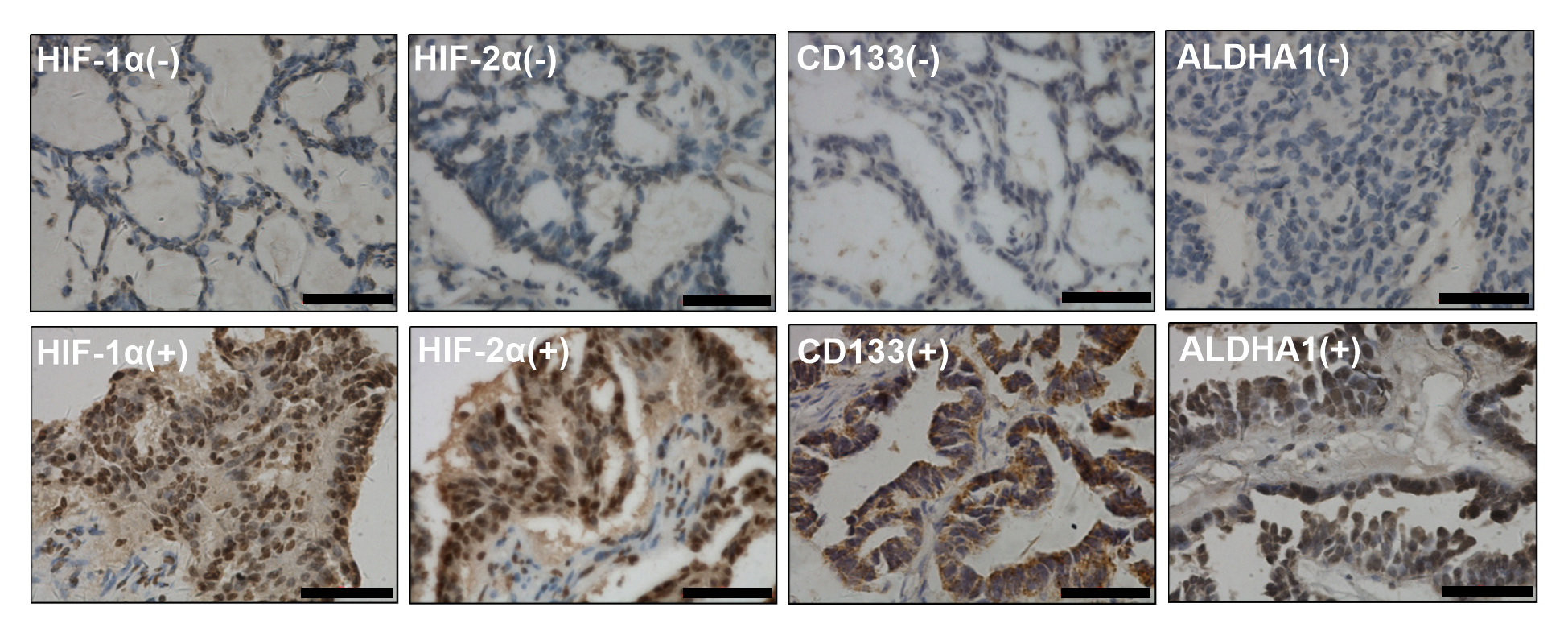

Supplement: Supplementary file 3 — Fig. S3. Representative pictures of the protein expression of HIF‐1α‐negative (HIF‐1α(‐)), HIF‐1α‐positive (HIF‐1α(+)), HIF‐2α‐negative (HIF‐2α(‐)), HIF‐2α‐positive (HIF‐2α(+)), CD133‐negative (CD133(‐)), CD133‐positive (CD133(+)), ALDHA1‐negative (ALDHA1(‐)), and ALDHA1‐positive (ALDHA1(+)) staining in 115 ovarian tumor tissues using immunohistochemistry. (Related to Fig. 2). [file MOL2-13-403-s003.tif]

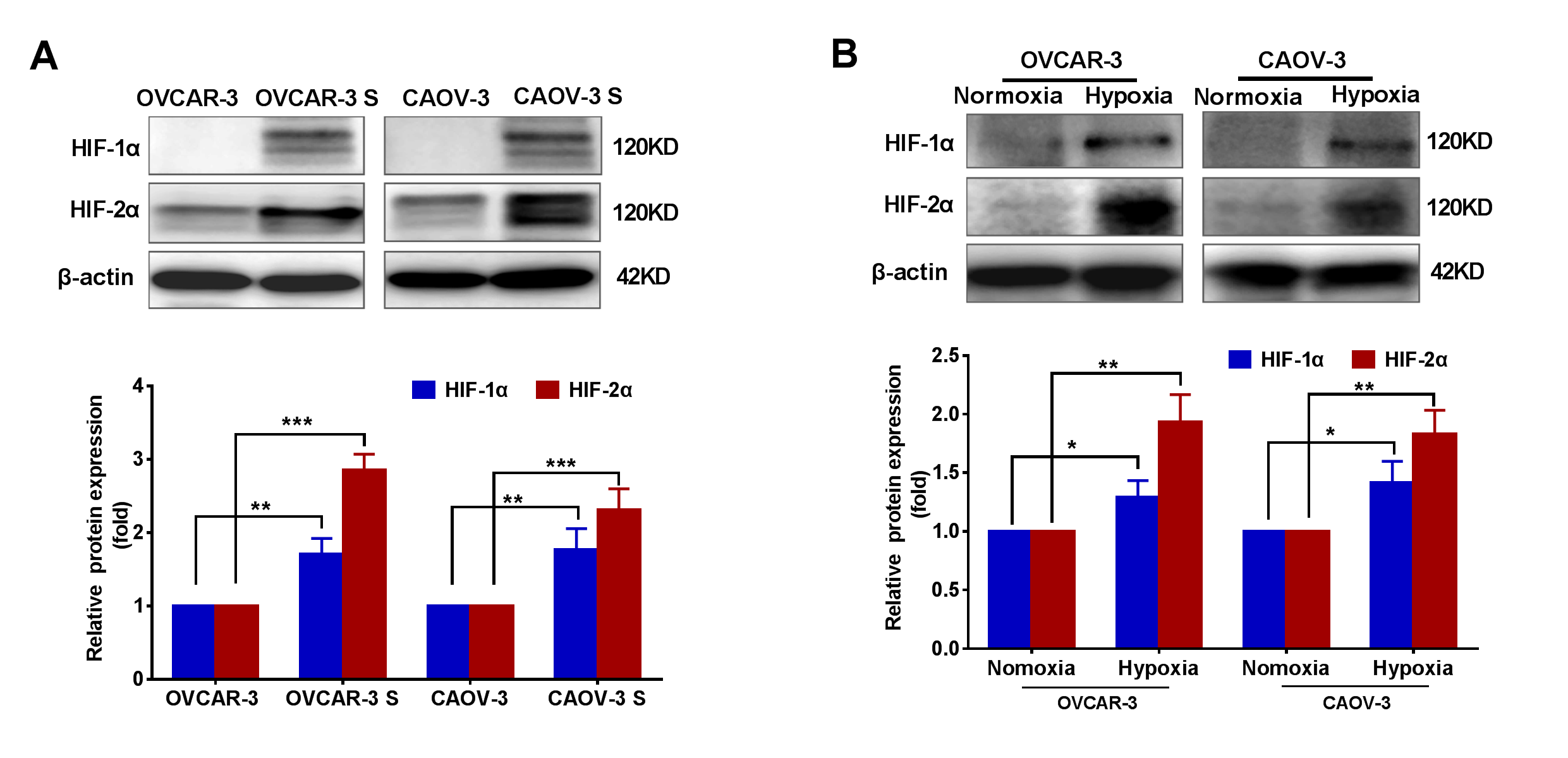

Supplement: Supplementary file 4 — Fig. S4. The expression changes of HIF‐1α or HIF‐2α in OVCAR‐3 S and CAOV‐3 S cells and hypoxia‐treated OVCAR‐3 and CAOV‐3 cells. [file MOL2-13-403-s004.tif]

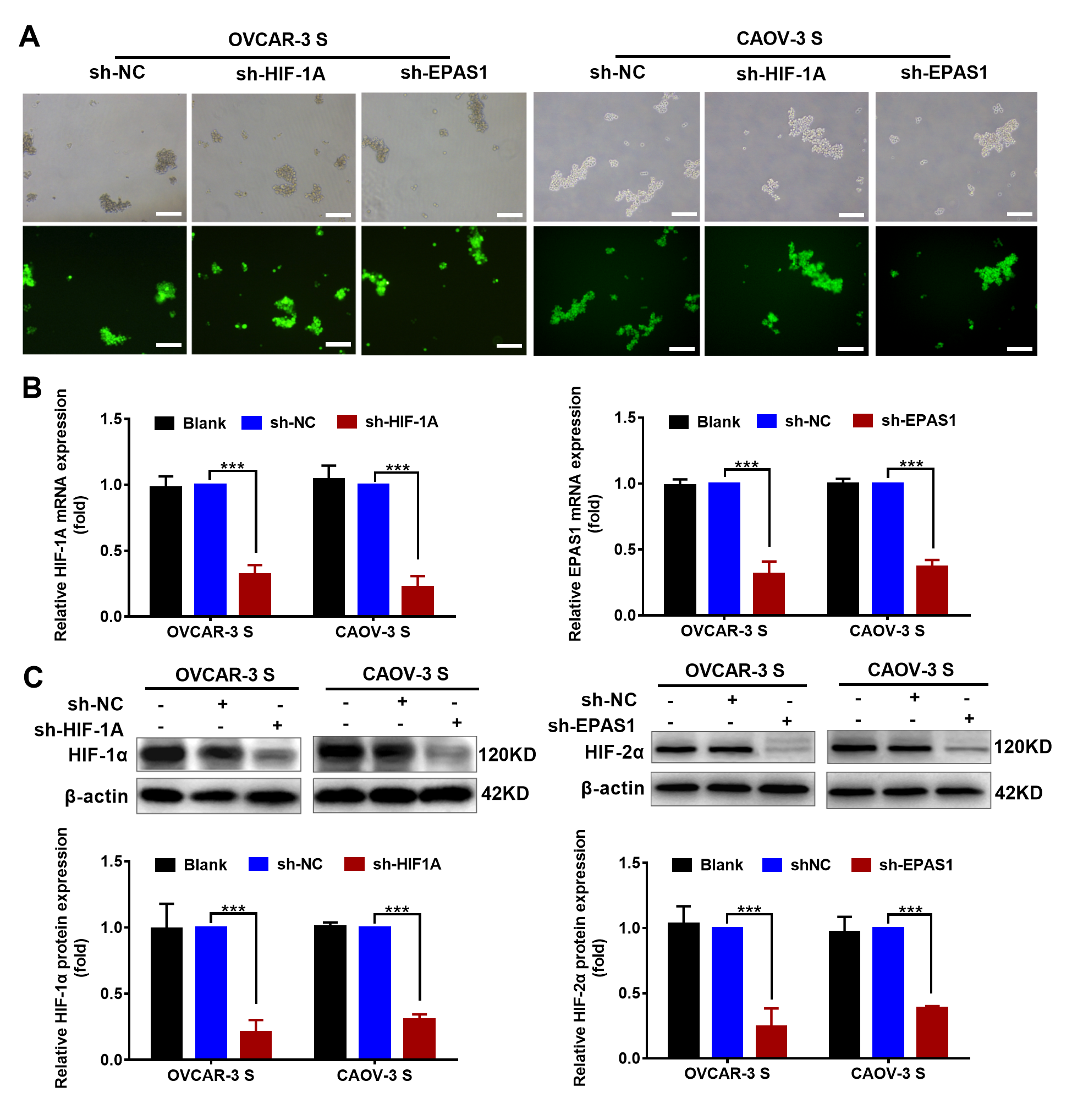

Supplement: Supplementary file 5 — Fig. S5. The transduction efficiency of OVCAR‐3 S and CAOV‐3 S cells with silenced HIF‐1α or HIF‐2α. [file MOL2-13-403-s005.tif]

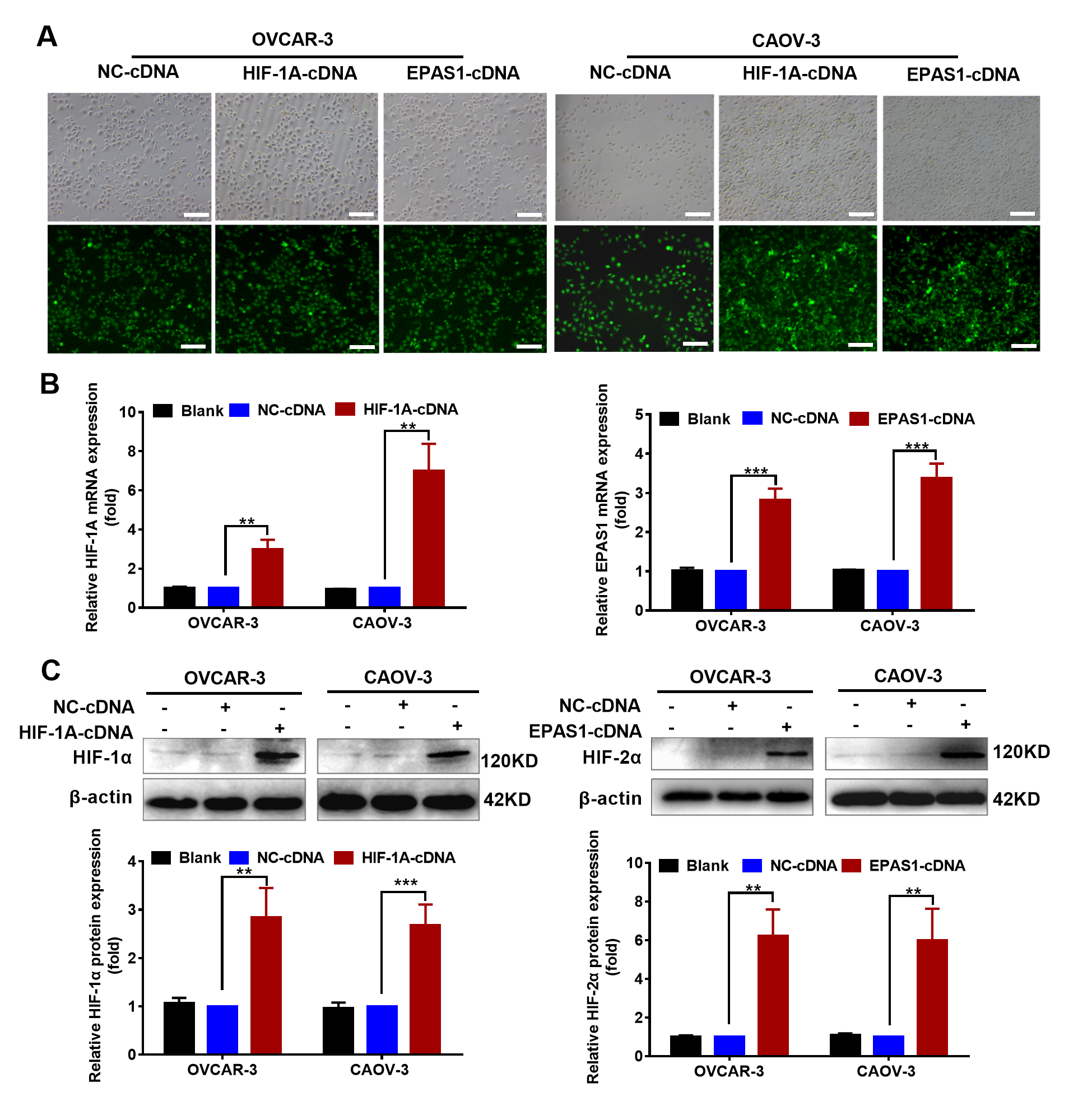

Supplement: Supplementary file 6 — Fig. S6. The transduction efficiency of OVCAR‐3 and CAOV‐3 cells overexpressing HIF‐1α or HIF‐2α. [file MOL2-13-403-s006.tif]

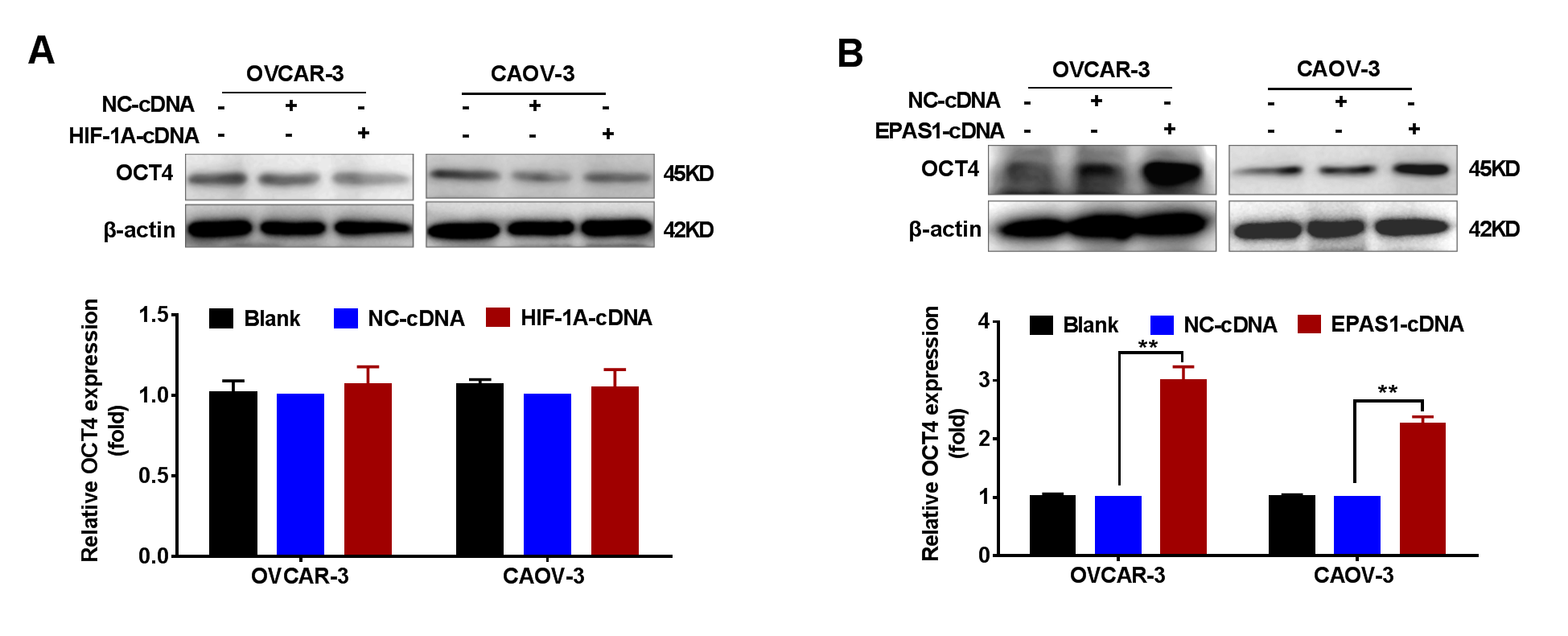

Supplement: Supplementary file 7 — Fig. S7. The effects of overexpression of HIF‐1α or HIF‐2α on the protein expression of OCT4 in OVCAR‐3 and CAOV‐3 cells. [file MOL2-13-403-s007.tif]

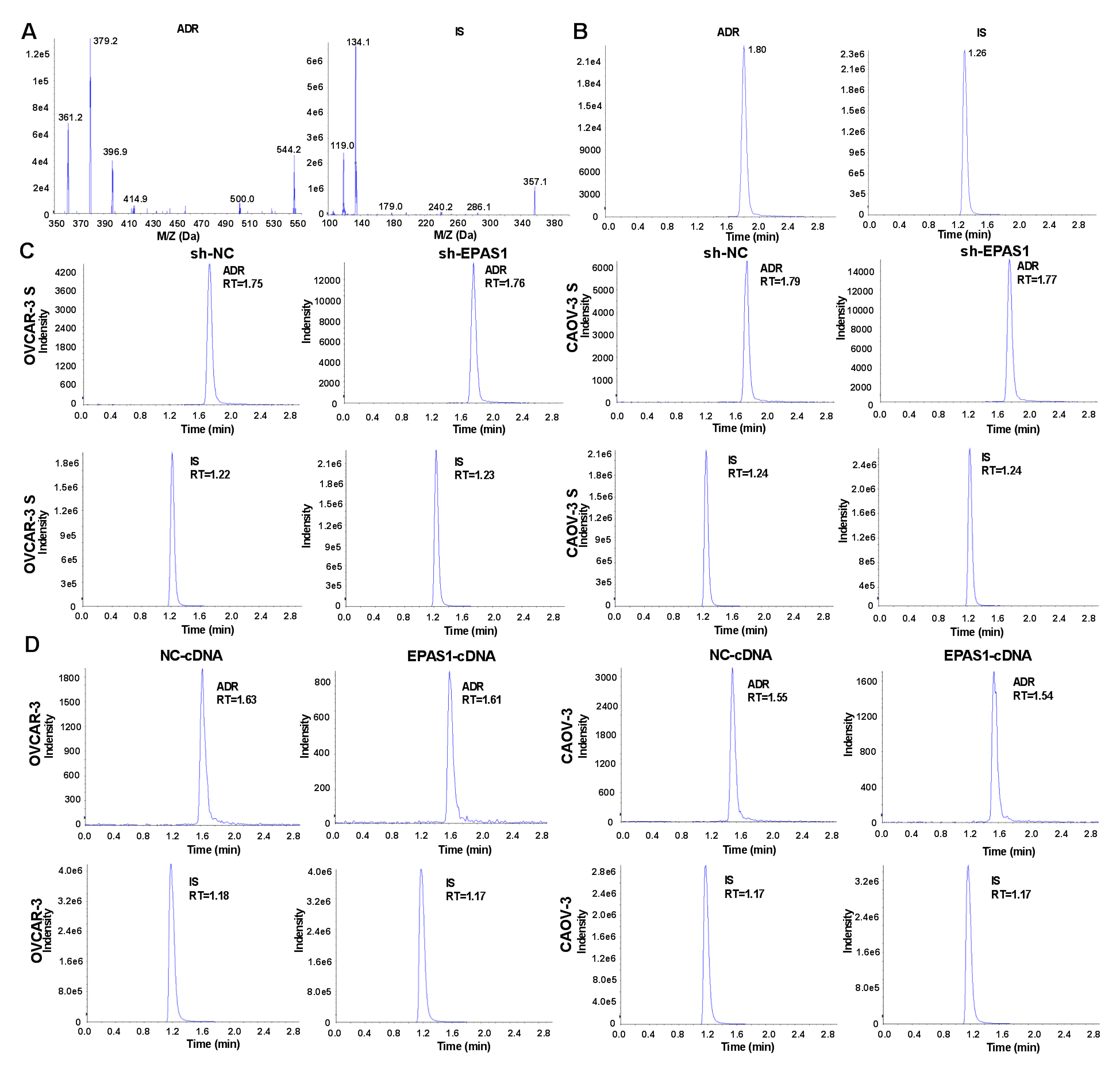

Supplement: Supplementary file 8 — Fig. S8. The effects of the HIF‐2α on the intracellular accumulation of ADR in ovarian cancer cells by mass spectrometry. [file MOL2-13-403-s008.tif]

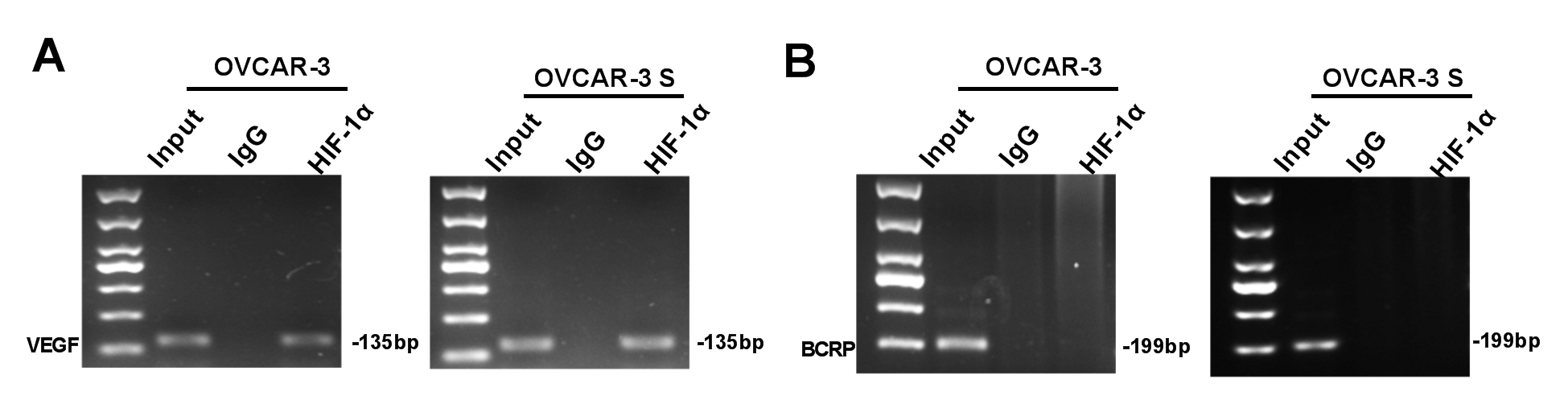

Supplement: Supplementary file 9 — Fig. S9. The determination of HIF‐1α binding to the VEGF and BCRP promoter. [file MOL2-13-403-s009.tif]

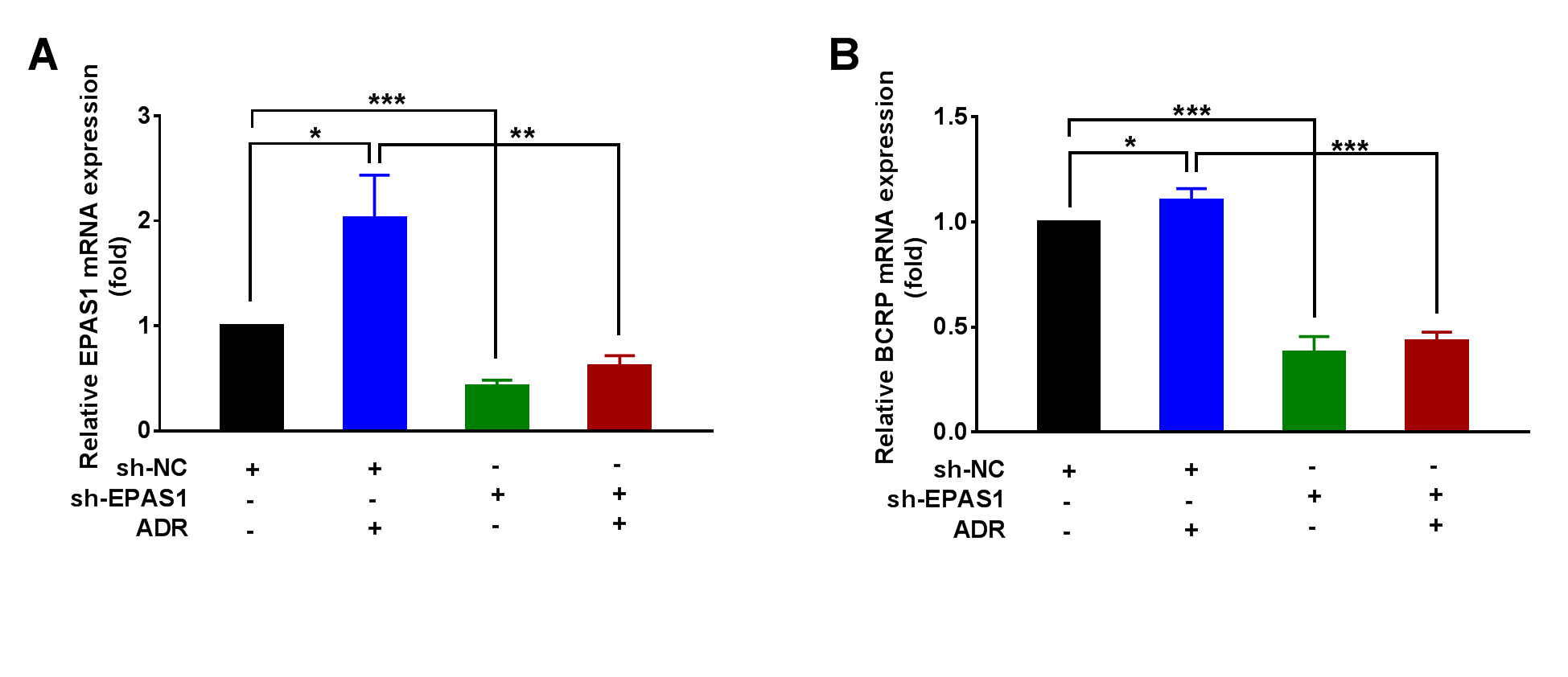

Supplement: Supplementary file 10 — Fig. S10. The effects of silencing HIF‐2α on the expression of BCRP in OCSCs xenograft mice. [file MOL2-13-403-s010.tif]
