# Supplementary material for: Hypoxia‐inducible factor‐2α directly promotes BCRP expression and mediates the resistance of ovarian cancer stem cells to adriamycin
Source: Mol Oncol. 2019 Jan 14;13(2):403–21. doi: 10.1002/1878-0261.12419 (PMC6360369; doi:10.1002/1878-0261.12419)
Supplement: Supplementary file 11 — Table S1. Clinicopathological features of 115 ovarian cancer patients. [file MOL2-13-403-s011.docx]

**Supplementary Table S1. Clinicopathological features of 115 ovarian cancer patients.**

| Features | Categories | Number | % |
| --- | --- | --- | --- |
| Age | ≤50 years | 77 | 67.0 |
|  | >50 years | 38 | 33.0 |
| Menopausal status | Premenopausal | 85 | 73.9 |
|  | Postmenopausal | 30 | 26.1 |
| Histological type | Serous | 101 | 87.8 |
|  | Mucinous | 6 | 5.2 |
|  | Clear cell | 4 | 3.5 |
|  | Endometrioid | 4 | 3.5 |
| FIGO stage | I- II | 61 | 53.0 |
|  | III-Ⅳ | 54 | 47.0 |
| Lymph node metastasis | No | 96 | 83.5 |
|  | Yes | 19 | 16.5 |
| Pathologic grade | Well differentiated | 35 | 30.4 |
|  | Moderately differentiated | 68 | 59.1 |
|  | Poorly differentiated | 12 | 10.5 |
