# Supplementary material for: Hypoxia‐inducible factor‐2α directly promotes BCRP expression and mediates the resistance of ovarian cancer stem cells to adriamycin
Source: Mol Oncol. 2019 Jan 14;13(2):403–21. doi: 10.1002/1878-0261.12419 (PMC6360369; doi:10.1002/1878-0261.12419)
Supplement: Supplementary file 12 [file MOL2-13-403-s012.docx]

**Supporting Information**

**Supplementary Figure S1. OVCAR-3 S and CAOV-3 S cells possess OCSC-like properties.** (Related to Figure 1) (A) Statistical analysis of the percentage of CD133-positive cells and ALDH-positive cells in OVCAR-3 vs. OVCAR-3 S cells and CAOV-3 vs. CAOV-3 S cells from flow cytometry. Data are presented as the mean ± SD from three independent experiments. **P*<0.05, ***P*<0.01. (B) OVCAR-3/OVCAR-3 S and CAOV-3/ CAOV-3 S cells were grown in soft agar. The colony numbers were counted manually under a microscope after 21 days of culture. Data are presented as the mean ± SD from three independent experiments. ****P*<0.001. (C) Invasion ability in OVCAR-3 vs. OVCAR-3 S cells and CAOV-3 vs. CAOV-3 S cells was measured using transwell invasion assays. Cells that invaded into the lower transwell chambers were counted. The number of OVCAR-3 or CAOV-3 cells migrating to the lower chambers was set as 1. Data are presented as the mean ± SD from three independent experiments. ***P*<0.01. (D) OVCAR-3 S and CAOV-3 S cells were cultured in DMEM medium with 10% FBS for 0 to 72 h.

**Supplementary Figure S2. Ovarian cancer sphere-forming cells, OVCAR-3 S and CAOV-3 S, are resistant to chemotherapeutic drugs.** (Related to Figure 1) Cell survival rate was analyzed by CCK-8 assays 48 hours after OVCAR-3 vs. OVCAR-3 S cells and CAOV-3 vs. CAOV-3 S cells were treated with different concentrations of (A) mitoxantrone (MX), (B) paclitaxel (PTX), (C) etoposide (VP-16), or (D) cisplatin (DDP). Data are from three independent experiments.

**Supplementary Figure S3.** Representative pictures of the protein expression of HIF-1α-negative (HIF-1α(-)), HIF-1α-positive (HIF-1α(+)), HIF-2α-negative (HIF-2α(-)), HIF-2α-positive (HIF-2α(+)), CD133-negative (CD133(-)), CD133-positive (CD133(+)), ALDHA1-negative (ALDHA1(-)), and ALDHA1-positive (ALDHA1(+)) staining in 115 ovarian tumor tissues using immunohistochemistry. (Related to Figure 2). Scale bars: 50 µm.

**Supplementary Figure S4. The expression changes of HIF-1α or HIF-2α** **in** **OVCAR-3 S and CAOV-3 S cells and hypoxia-treated OVCAR-3 and CAOV-3 cells.** (Related to Figure 2). (A) Protein expression levels of HIF-1α and HIF-2α were analyzed by western blot in OVCAR-3 S and CAOV-3 S cells versus their parental cells. β-actin was used as a loading control. The expression level of HIF-1α or HIF-2α was normalized to that of β-actin. The HIF-1α or HIF-2α expression level in OVCAR-3 cells or CAOV-3 cells were set as 1. Data are presented as mean ± SD from three independent experiments. ***P*<0.01, ****P*<0.001. (B) Protein expression levels of HIF-1α and HIF-2α were analyzed by western blot in OVCAR-3 and CAOV-3 cells under hypoxia (1% O_2_) culture for 48 hours. β-actin was used as a loading control. The expression levels of HIF-1α or HIF-2α were normalized to that of β-actin. The HIF-1α or HIF-2α expression levels in OVCAR-3 cells or CAOV-3 cells under normoxia culture for 48 hours were set as 1. Data are presented as the mean ± SD from three independent experiments. * *P*<0.05, ** *P*<0.01.

**Supplementary Figure S5. The transduction efficiency of OVCAR-3 S and CAOV-3 S cells with silenced *HIF-1α* or *HIF-2α*.** (Related to Figure 3). OVCAR-3 S and CAOV-3 S cells were transduced for 48 hours under hypoxic conditions (1% O_2_) with shRNAs against *HIF-1A* (sh-*HIF-1A*), *EPAS1* (sh-*EPAS1*), or a negative control (sh-NC). (A) Green fluorescent protein (GFP) was observed in transduced cells under fluorescence microscope. (B) qRT-PCR and western blot (C) were performed to test the mRNA and protein expression of HIF-1α or HIF-2α in the cells after transduction. β-actin was used as an endogenous control. Relative mRNA and protein expression of HIF-1α or HIF-2α was normalized to the expression in sh-NC samples. Data are presented as the mean ± SD from three independent experiments. *** *P*<0.001. Scale bars: 200 µm.

**Supplementary Figure S6. The transduction efficiency of OVCAR-3 and CAOV-3 cells over-expressing HIF-1α or HIF-2α.** (Related to Figure 3). OVCAR-3 and CAOV-3 cells were transduced with cDNAs encoding *HIF-1A* (*HIF-1A*-cDNA), *EPAS1* (*EPAS1*-cDNA), or a negative control (NC-cDNA). (A) Green fluorescent protein (GFP) was observed in the transduced cells using a fluorescence microscope. qRT-PCR (B) and western blot (C) were performed to analyze mRNA and protein expression of HIF-1α or HIF-2α in cells after transduction. β-actin was used as an endogenous control. Relative mRNA and protein expression of HIF-1α or HIF-2α was normalized to the expression in NC-cDNA samples. Data are presented as the mean ± SD from three independent experiments. * *P*<0.05, ** *P*<0.01, *** *P*<0.001. Scale bars: 200 µm.

**Supplementary Figure S7. The effects of overexpression of HIF-1α or HIF-2α on the protein expression of OCT4 in OVCAR-3 and CAOV-3 cells.** (Related to Figure 3). (A) Protein expression changes of OCT4 were analyzed by western blot in *HIF-1A*-cDNA-transduced OVCAR-3 and CAOV-3 cells. β-actin was used as a loading control. The expression level of OCT4 was normalized to that of β-actin. The OCT4 expression levels in *NC*-cDNA-transduced OVCAR-3 cells or CAOV-3 cells were set as 1. Data are presented as mean ± SD from three independent experiments. (B) Protein expression changes of OCT4 were analyzed by western blot in *EPAS1*-cDNA-transduced OVCAR-3 and CAOV-3 cells. β-actin was used as a loading control. The expression level of OCT4 was normalized to that of β-actin. The OCT4 expression levels in *NC*-cDNA-transduced OVCAR-3 cells or CAOV-3 cells were set as 1. Data are presented as mean ± SD from three independent experiments. ** *P*<0.01.

**Supplementary Figure S8. The effects of the HIF-2α on the intracellular accumulation of ADR in ovarian cancer cells by mass spectrometry.** **(Related to Figure 4).** (A) Mass spectrograms for ADR and pioglitazone, used as an internal standard (IS) for blank samples. (B) Chromatograms for ADR and IS of blank samples. (C) Representative chromatograms for the intracellular accumulation of ADR and pioglitazone, used as an internal standard (IS), in OVCAR-3 S and CAOV-3 S cells transduced with sh-*HIF-2α* or sh-NC lentivirus. (D) Representative chromatograms for the intracellular accumulation of ADR and IS in the OVCAR-3 and CAOV-3 cells transduced with *EPAS1*-cDNA or NC-cDNA.

**Supplementary Figure S9.** **The determination of HIF-1α binding to the VEGF and BCRP promoter. (Related to Figure 5).** (A) ChIP assays were performed to verify HIF-1α binding to the *VEGF* gene in OVCAR-3 and OVCAR-3 S cells. (B) ChIP assays were performed to test HIF-1α binding to the *BCRP* gene in OVCAR-3 and OVCAR-3 S cells.

**Supplementary Figure S10.** The effects of silencing *HIF-2α* on the expression of BCRP in OCSCs xenograft mice. **(Related to Figure 6).** Relative mRNA expression of (A) EPAS1 and (B) BCRP normalized to those of β-actin were analyzed in xenograft tumors by qRT-PCR. The expression level of EPAS1/BCRP in the xenograft tumors transduced with sh-NC and without ADR treatment were set as 1. Data are presented as the mean ± SD from three independent experiments. **P*<0.05, ***P*<0.01, ****P*<0.001.
